# Mini-GAGR, an intranasally applied polysaccharide, activates the neuronal Nrf2-mediated antioxidant defense system

**DOI:** 10.1074/jbc.RA117.001245

**Published:** 2018-10-03

**Authors:** Kelsey Murphy, Killian Llewellyn, Samuel Wakser, Josef Pontasch, Natasha Samanich, Matthew Flemer, Kenneth Hensley, Dong-Shik Kim, Joshua Park

**Affiliations:** From the Departments of ‡ Neurosciences and; § Pathology, College of Medicine and Life Sciences, University of Toledo, Toledo, Ohio 43614 and; the ¶ Department of Chemical Engineering, College of Engineering, University of Toledo, Toledo, Ohio 43607

**Keywords:** Alzheimer disease, antioxidant, nuclear factor 2 (erythroid-derived 2-like factor) (NFE2L2) (Nrf2), polysaccharide, neurodegeneration, mini-GAGR, BBB-bypassing polysaccharide, antioxidant enzymes, amyloid, tau, oxidative stress

## Abstract

Oxidative stress triggers and exacerbates neurodegeneration in Alzheimer's disease (AD). Various antioxidants reduce oxidative stress, but these agents have little efficacy due to poor blood–brain barrier (BBB) permeability. Additionally, single-modal antioxidants are easily overwhelmed by global oxidative stress. Activating nuclear factor erythroid 2 (NF-E2)-related factor 2 (Nrf2) and its downstream antioxidant system are considered very effective for reducing global oxidative stress. Thus far, only a few BBB-permeable agents activate the Nrf2-dependent antioxidant system. Here, we discovered a BBB-bypassing Nrf2-activating polysaccharide that may attenuate AD pathogenesis. Mini-GAGR, a 0.7-kDa cleavage product of low-acyl gellan gum, increased the levels and activities of Nrf2-dependent antioxidant enzymes, decreased reactive oxygen species (ROS) under oxidative stress in mouse cortical neurons, and robustly protected mitochondria from oxidative insults. Moreover, mini-GAGR increased the nuclear localization and transcriptional activity of Nrf2 similarly to known Nrf2 activators. Mechanistically, mini-GAGR increased the dissociation of Nrf2 from its inhibitor, Kelch-like ECH-associated protein 1 (Keap1), and induced phosphorylation and nuclear translocation of Nrf2 in a protein kinase C (PKC)- and fibroblast growth factor receptor (FGFR1)-dependent manner. Finally, 20-day intranasal treatment of 3xTg-AD mice with 100 nmol of mini-GAGR increased nuclear p-Nrf2 and growth-associated protein 43 (GAP43) levels in hippocampal neurons, reduced p-tau and β-amyloid (Aβ) peptide–stained neurons, and improved memory. The BBB-bypassing Nrf2-activating polysaccharide reported here may be effective in reducing oxidative stress and neurodegeneration in AD.

## Introduction

Alzheimer's disease (AD)^3^ is a devastating neurodegenerative disease that impairs memory and causes cognitive defect. AD affects 35.6 million people worldwide and its incidence is expected to increase to 115 million people by 2050. Despite the upcoming surge of AD frequency, there is still no effective treatment.

Oxidative stress is thought to be a major trigger of AD, as we reviewed recently (1). Usually, aging gradually reduces endogenous antioxidant capacity, causing surrender to overwhelming oxidative species. The loss of antioxidant capacity occurs even faster in AD brain (2). Therefore, reducing oxidative stress is key to slowing neurodegeneration in AD brain. However, current antioxidant supplement treatments are ineffective in controlling global oxidative stress in the brain because of poor blood–brain barrier (BBB) permeability and single-modal targeting. One way to control global oxidative stress is to activate nuclear factor erythroid 2 (NF-E2)-related factor 2 (Nrf2), the main transcriptional factor of a majority of endogenous antioxidant and detoxicating enzymes (3). Currently, there are a few BBB-permeable Nrf2 activators that show some efficacy in treating brain diseases, including dimethyl fumarate (DMF) (4) and CDDO-TFEA (5, 6).

In the brains of AD model mice, the Nrf2-antioxidant response element (ARE) system appears to be less active compared with WT brains (7). In the hippocampus of AD model mice, most Nrf2 was excluded from nuclei (8). In the other AD model mice, Nrf2 knockout exacerbated AD progression, suggesting that Nrf2 acts as a sentinel to reduce further AD advancement (9). Conversely, Nrf2 activation attenuated effects on neurodegeneration in several AD model mice (7, 10–13). When activated, Nrf2 is released from its repressor Kelch-like ECH-associated protein 1 (Keap1) and enters the nucleus (14), where it binds to and activates ARE in the promoters of the genes that encode proteins involved in iron homeostasis (HO-1 and ferritin), redox regulation (superoxide dismutase (SOD), catalase (CAT), peroxiredoxin (Prx), sulfiredoxin (Srx), and thioredoxin (Trx)), GSH synthesis (GSH reductase (GR), GSH peroxidase (GPx), GSH cysteine ligase regulatory and modulatory subunits, γ-glutamyl cysteine synthetase (γ-GCS)), and quinone recycling (NAD(P)H:quinoneoxidoreductase 1 (NQO1)) (15, 16). The antioxidant enzymes, then, scavenge various intracellular oxidative radicals. For example, HO-1 works with NADPH cytochrome P450 reductase to convert heme to biliverdin, which is converted to bilirubin by biliverdin reductases (17). Both biliverdin and bilirubin have strong antioxidant and anti-inflammatory effects (18). CAT mediates the conversion of H_2_O_2_ to water (19), Trx and the GSH system detoxicate ONOO^−^ (20), SOD converts O_2_^˙̄^ to H_2_O_2_ (21), and GPx reduces H_2_O_2_ to water (22, 23). GSH provides an electron to GPx for H_2_O_2_ reduction and then is recycled by GR and NADPH/H^+^. GSH is also generated from glutamate, cysteine, and glycine by γ-GCS and GSH synthetase (GS). Glutaredoxin (Grx) reduces protein disulfides (GSSG) via a disulfide exchange reaction with the expense of GSH to GSSG (22). GSSG is then reduced to GSH by GR. NQO1 is involved in two-electron reduction of reactive quinones utilizing NADH or NADPH as a reducing co-factor (24). Thus, activating Nrf2 is expected to increase and deploy the above antioxidant enzymes to scavenge various oxidative radicals and increase the endogenous antioxidant, GSH.

Oxidative stress also causes mitochondrial dysfunction, thus facilitating AD pathogenesis (25). Mitochondrial dysfunction begins with the uncoupling of mitochondrial electron transport chain and the depolarization of mitochondrial membrane potential (MMP), which results in reactive oxygen species (ROS) production and ATP depletion (26). Without protecting the mitochondria, it is difficult to reduce oxidative stress and slow neurodegeneration. Thus, to reduce global oxidative stress, mitochondria and MMP should be protected.

We discovered a BBB-bypassing Nrf2 activator in mini-GAGR, the 0.7-kDa cleavage product of low-acyl gellan gum that has few side effects in humans (27) and is registered as a human food additive (Food and Drug Administration 21 CFR 172.665). Like midi-GAGR, the 4.7-kDa cleavage product of low-acyl gellan gum (28, 29), mini-GAGR has a good BBB-bypassing ability. Given that mini-GAGR and midi-GAGR share the same repeating unit, d-Glc[β1→4]d-GlcA[β1→4]d-Glc[β1→4]l-Rha [α1→3], they are expected to have similar physiological effects with respect to cellular receptor interactions with fibroblast growth factor receptor 1 (FGFR1) that was found to interact with midi-GAGR in our previous study (28). The only difference between mini-GAGR and midi-GAGR may be their ability to diffuse through the brain matrix in an *in vivo* environment. Because of its smaller size, mini-GAGR may diffuse much faster and deeper through the matrix than midi-GAGR. As such, mini-GAGR may have a pharmacological advantage over midi-GAGR regarding its ability to reach the hippocampus, which is why we focused on mini-GAGR in this study.

To examine an *in vivo* effect of mini-GAGR in an AD animal model, we used 3xTg-AD mice. 3xTg-AD mice that are designed to develop amyloid plaques and neurofibrillary tangles (30) harbor two familial AD mutations, APP_swe_ and PS1_M146V_, and the tau_P301L_ mutation found in frontotemporal dementia (31, 32) and are used to examine potential AD therapies (33, 34). Around 12 months of age, 3xTg-AD mice show defects in spatial reference learning and memory in the Barnes maze (35, 36) and other behavioral tests (37, 38). Aβ peptide is detected in the cortex and hippocampus from 6 months of age (39), whereas tau hyperphosphorylation is detected in the hippocampus around 12 months of age (31). Female 3xTg-AD mice develop a higher Aβ burden and exhibit worse cognitive performances compared with male 3xTg-AD mice (40–43). Importantly, the brains of 12-month-old 3xTg-AD mice lose their stance in antioxidant defense, resulting in increased intracerebral oxidative stress and accompanying oxidative damage (44). Taken together, 12-month-old 3xTg-AD mice are a good model to test the efficacy of a drug on both antioxidant system and AD pathology.

Based on our current study, the neuroprotective effect of mini-GAGR appears to be mediated by its ability to activate Nrf2 and its downstream antioxidant enzymes. Here, we demonstrate the novel Nrf2-activating action of the BBB-bypassing polysaccharide, mini-GAGR.

## Results

### Mini-GAGR increases protein levels of antioxidant enzymes in mouse cortical neurons

**Figure 1.**
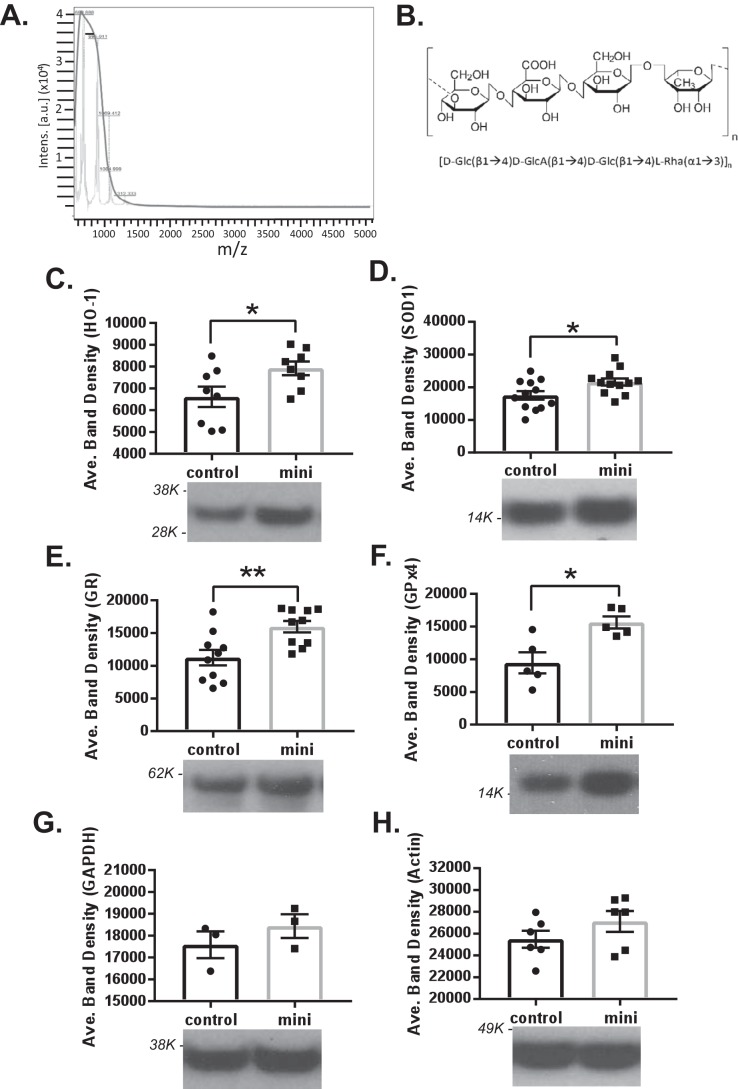
**Mass spectrometry of mini-GAGR and its effect on antioxidant enzyme proteins in mouse cortical neurons.** *A*, mass spectrum of mini-GAGR. MALDI-MS shows the molecular mass (∼720 Da) of mini-GAGR. *B*, basic structure of mini-GAGR. *C–H*, mouse cortical neurons (E17, DIV11–14) were treated with either vehicle (control) or 1 μm mini-GAGR (*mini*) for 48 h and processed for immunoblotting using antibodies to antioxidant enzymes and GAPDH and β-actin (loading control). The band densities of the proteins were quantified by ImageJ to obtain average band density ± S.E. (*error bars*) for the bar graphs. *C*, HO1 (32 kDa) (*p* = 0.0364, *n* = 8 different embryo batches); *D*, SOD1 (16 kDa) (*p* = 0.0243, *n* = 12 different embryo batches); *E*, GR (60 kDa) (*p* = 0.00515, *n* = 10 different embryo batches); *F*, GPx4 (21 kDa) (*p* = 0.0103, *n* = 5 different embryo batches) compared with control. There were no statistically significantly differences in the protein levels of GAPDH (37 kDa) and β-actin (45 kDa) in mini-GAGR–treated neurons compared with control (*, *p* < 0.05; **, *p* < 0.01; unpaired *t* test, two-tailed). Data are expressed as mean ± S.E. Each molecular mass marker is marked *beside* each blot (*e.g. 98K* indicates 98,000 Da).

Our previous study demonstrated that midi-GAGR, a 4.7-kDa cleavage product of low-acyl gellan gum, exerted a strong neuroprotective effect on mouse cortical neurons under oxidative stress caused by the pathological concentrations of H_2_O_2_, 4-hydroxynonenal (4HNE), and Aβ_42_ peptide found in AD brains (28). The neurotrophic effect of midi-GAGR appears to partly contribute to its neuroprotective effect (28), although it might not be sufficient enough to offer neuronal protection against oxidative stress. Given that antioxidant enzymes are the major endogenous defense system to scavenge free reactive radicals (1), it is possible that antioxidant enzymes might be increased by the low-acyl gellan gum cleavage product, mini-GAGR. To examine this possibility, we tested the effect of mini-GAGR on the protein levels of several major antioxidant enzymes in mouse embryonic cortical neurons (E17, DIV11–14). Mini-GAGR is a 0.7-kDa cleavage product of low-acyl gellan gum that shares the same repeating unit, (d-Glc[β1→4]d-GlcA[β1→4]DGlc[β1→4] l-Rha[α1→3])*_n_*, as midi-GAGR (mini-GAGR, *n* = 1; midi-GAGR, *n* = 4). Mini-GAGR is expected to have a pharmacological advantage (greater diffusibility) over midi-GAGR, although with an extent of physiological effect (*e.g.* neurotrophic effect) similar to that of midi-GAGR (28). We generated mini-GAGR by 72-h digestion with α(1→3) glycosidase and measured its molecular weight using a mass spectrometer (Fig. 1*A*). The apex of the bell curve, shown with the *black solid line*, indicates that most of the oligosaccharide fragments in mini-GAGR MS have a peak intensity distribution centered around a smaller molecular mass (∼720 Da) according to MS in the α-cyano-4-hydroxycinnamic acid matrix. Because MALDI-MS measures only a powder form of chemical, it could not measure the molecular weight of vehicle, water. The chemical structure of mini-GAGR is shown in Fig. 1*B*.

We examined the protein levels of two major ROS-scavenging enzymes, HO-1 and SOD1, and two major GSH-generating enzymes, GR and GPx4, in neurons that were treated with either vehicle (water) or mini-GAGR. In addition to the antioxidant enzymes, we examined protein levels of loading controls, glyceraldehyde 3-phosphate dehydrogenase (GAPDH) and β-actin, in neurons treated with either vehicle or 1 μm mini-GAGR. After 24 h, there were only slight increases in the protein levels of the enzymes (data not shown). Conversely, after 48 h, we could detect noticeable increases in protein levels. Compared with control (neurons treated with vehicle), the protein levels of HO-1 and SOD1 were significantly increased (Fig. 1, *C* and *D*). The average band densities of each protein band were measured using ImageJ software to obtain mean ± S.E. for bar graphs (Fig. 1, *C–H*). HO-1 was increased by ∼1.2-fold (6613.25 ± 466.81 for control *versus* 7920.19 ± 318.28 for mini-GAGR (*mini*)) (Fig. 1*C*). SOD1 was increased by ∼1.2-fold (17,520.89 ± 1308.07 for control *versus* 21,616.41 ± 1075.90 for mini-GAGR (*mini*)) (Fig. 1*D*). GR was increased by ∼1.4-fold (11,249.38 ± 1191.78 for control *versus* 15,960.72 ± 877.48 for mini-GAGR (*mini*)) (Fig. 1*E*). GPx4 was increased by ∼1.6-fold (9441.55 ± 1616.52 for control *versus* 15,636.90 ± 918.38 for mini-GAGR (*mini*)) (Fig. 1*F*). On the other hand, the loading controls, GAPDH and β-actin, did not show any significant change between mini-GAGR and vehicle treatments (GAPDH: 17,581.18 ± 610.69 for control *versus* 18,435.36 ± 543.28 for mini-GAGR (*mini*); β-actin: 24,970.53 ± 833.06 for control *versus* 27,722.79 ± 1012.96 for mini-GAGR (*mini*)) (Fig. 1, *G* and *H*). Thus, it is clear that mini-GAGR treatment increases the protein levels of the major antioxidant enzymes, HO-1, SOD1, GR, and GPx4, in mouse cortical neurons.

### Mini-GAGR increases Nrf2 nuclear localization and transcriptional activity

**Figure 2.**
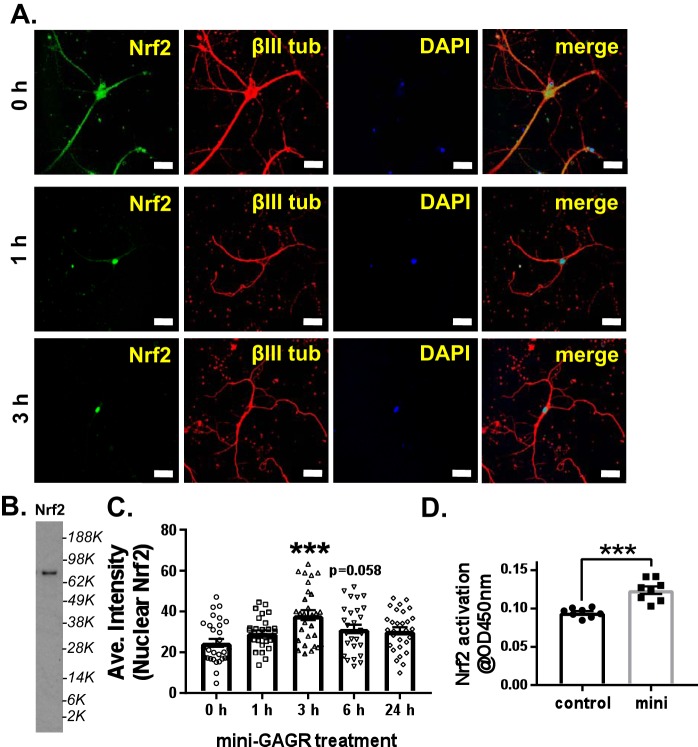
**Mini-GAGR increases the nuclear translocation and transcription factor activity of Nrf2.** *A*, mouse cortical neurons (E17, DIV11–14) were treated with 1 μm mini-GAGR for 0, 1, 3, 6, and 24 h and processed for immunocytochemistry with antibodies against Nrf2 (*green*; Alexa Fluor 488), βIII tubulin (*red*; Alexa Fluor 594), and DAPI (*blue*) (*scale bar*, 30 μm). *B*, the specificity of anti-Nrf2 antibody (80 kDa) was confirmed by immunoblotting of the cytosol of untreated mouse cortical neurons. *C*, the fluorescence intensities of nuclear Nrf2 in images were quantified using Metamorph to calculate the average intensity ± S.E. (*error bars*) of nuclear Nrf2 staining during the time course and used for bar graphs: compared with control, 3 h (*p* < 0.001, *n* = 30 cells) and 6 h (*p* = 0.058, *n* = 30 cells). (One-way ANOVA (F(4, 145) = 6.2641, *p* < 0.001) and Bonferroni's multiple-comparison test were used). *D*, the transcription factor activity of Nrf2 was measured 3 h after mini-GAGR treatment via absorbance at 450 nm for the bar graphs: control (0.094 ± 0.002, *n* = 8 different batches of embryos) and mini-GAGR (0.124 ± 0.005, *n* = 8 different embryo batches) (*p* < 0.001) (***, *p* < 0.001; unpaired *t* test, two-tailed).

Because Nrf2 is the major transcriptional factor for the expression of antioxidant enzymes (1), it is possible that mini-GAGR, which increases antioxidant enzymes, activates Nrf2 in mouse cortical neurons. First, we examined whether mini-GAGR induced the nuclear translocation of Nrf2 in mouse cortical neurons (E17, DIV11–14). Neurons were treated with 1 μm mini-GAGR for 0, 1, 3, 6, and 24 h; fixed in 3.7% paraformaldehyde; and stained with antibodies to Nrf2 and βIII tubulin along with DAPI. At 0 h, Nrf2 was distributed evenly between the nucleus and cytoplasm along neurites (Fig. 2*A*). At 1 h, the levels of Nrf2 in the nucleus appeared to be increased, whereas the intensity of Nrf2 in the nucleus reached a very noticeable level at 3 h (Fig. 2*A*). We confirmed the specificity of the anti-Nrf2 antibody used for immunostaining by additional immunoblotting using mouse neuron extracts (Fig. 2*B*). We quantified the fluorescence intensities of Nrf2 in the nuclei of neurons treated with mini-GAGR for different time periods using Metamorph software and calculated the mean ± S.E. of the intensities for bar graphs. At 3 h after the treatment with mini-GAGR, nuclear Nrf2 showed significantly higher intensity (38.17 ± 2.45) than that at 0 h (24.65 ± 1.91) (Fig. 2*C*). Thereafter, the nuclear levels of Nrf2 were reduced to slightly higher than that at 0 h (6 h, 31.44 ± 2.04; 24 h, 30.50 ± 1.77) (Fig. 2*C*). This result suggests that mini-GAGR treatment induces the nuclear translocation of Nrf2 in mouse cortical neurons.

Then we used a commercial kit (Abcam) to measure the transcriptional activity of Nrf2 in mouse cortical neurons treated with either control (vehicle) or 1 μm mini-GAGR for 3 h. Nuclei were harvested from hypotonically ruptured cells, and proteins were extracted from the nuclei in 10% Igepal CA-630 to obtain nuclear extracts. Then Nrf2 in the nuclear extracts was mixed with dsDNAs that contain Nrf2-binding sites, such as ARE. Unbound Nrf2 was washed out, and bound Nrf2 was detected using anti-Nrf2 horseradish peroxidase (HRP) antibody for the absorbance at 450 nm. As a result, neurons treated with mini-GAGR for 3 h showed higher Nrf2 activity than those with vehicle (0.094 ± 0.002 for control and 0.124 ± 0.005 for mini-GAGR; Fig. 2*D*). Thus, mini-GAGR induces the nuclear localization of Nrf2 and increases the transcriptional activity of Nrf2.

### Mini-GAGR activates Nrf2 similarly to known Nrf2 activators

**Figure 3.**
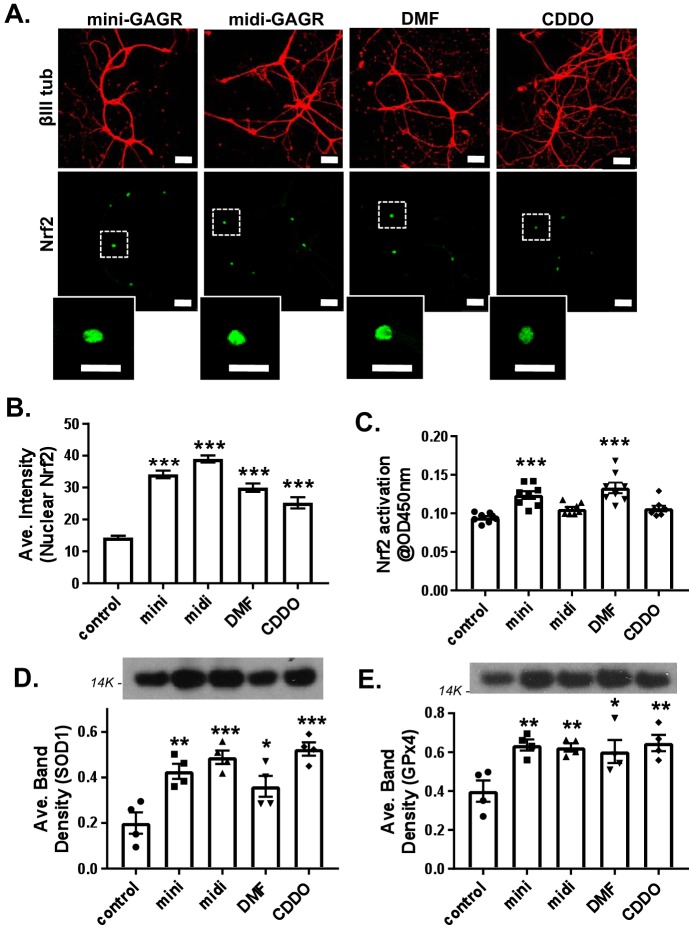
**Comparison of mini-GAGR with other Nrf2 activators.** *A*, mouse cortical neurons (E17, DIV11–14) were treated with vehicle (control), 1 μm mini-GAGR, 1 μm midi-GAGR, 6 μg/ml DMF, or 100 nm CDDO-TFEA for 3 h and processed for immunocytochemistry with antibodies against Nrf2 (*green*; Alexa Fluor 488), βIII tubulin (*red*; Alexa Fluor 594), and DAPI (not shown) (*scale bar*, 30 μm). The *insets* show each representative nuclear Nrf2 staining. *B*, the fluorescence intensities of nuclear Nrf2 in images were quantified using Metamorph to calculate the average intensity ± S.E. (*error bars*) for bar graphs (*n* = 76–80 nuclei): control *versus* mini-GAGR, midi-GAGR, DMF, and CDDO (*p* < 0.001) (*F* (4, 509) = 87.888, *p* < 0.001, ANOVA). (Of note, because the total number of measurements per condition was too large for a scatter plot, bar graphs were used instead.) *C*, the transcriptional factor activity of Nrf2 was measured via absorbance at 450 nm at 3 h after the treatment with vehicle (control), 1 μm mini-GAGR, 1 μm midi-GAGR, 6 μg/ml DMF, or 100 nm CDDO-TFEA. *Bar graphs*, control *versus* mini-GAGR and control *versus* DMF (*n* = 8 different embryo batches, *p* < 0.001) (F(4, 35) = 12.668, *p* < 0.001, ANOVA). *D* and *E*, mouse primary cortical neurons (E17, DIV11–14) were treated with vehicle (control), 1 μm mini-GAGR, 1 μm midi-GAGR, 6 μg/ml DMF, or 100 nm CDDO-TFEA for 48 h and processed for immunoblotting using antibodies to SOD1 (16 kDa) and GPx4 (21 kDa). The band densities of the proteins were quantified by ImageJ to obtain average band density ± S.E. for the bar graphs. *D*, SOD1 (*n* = 4 different embryo batches): control *versus* mini-GAGR (*p* = 0.003), control *versus* midi (*p* < 0.001), control *versus* DMF (*p* = 0.034), and control *versus* CDDO-TFEA (*p* < 0.001) (F(4, 15) = 11.566, *p* < 0.001, ANOVA). *E*, GPx4 (*n* = 4 different embryo batches): control *versus* mini-GAGR (*p* = 0.007), control *versus* midi-GAGR (*p* = 0.010), control *versus* DMF (*p* = 0.020), control *versus* CDDO-TFEA (*p* = 0.005) (F(4, 15) = 5.5698, *p* = 0.006, ANOVA). (*, *p* < 0.05; **, *p* < 0.01; ***, *p* < 0.001, one-way ANOVA and Bonferroni's multiple-comparison test.) Data are expressed as mean ± S.E.

We wondered whether mini-GAGR activates Nrf2 in a similar way to known Nrf2 activators (*e.g.* DMF (4) and CDDO-TFEA (45, 46)). Thus, we compared the extent to which mini-GAGR increased the nuclear localization and activation of Nrf2 and the expression of two antioxidant enzymes, SOD1 and GPx4, with those increased by DMF and CDDO-TFEA. We also compared these effects with midi-GAGR to know whether the larger GAGR unit containing four repeating units has the same effect as mini-GAGR. First, mouse cortical neurons (E17, DIV11–14) were treated with vehicle (control), 1 μm mini-GAGR, 1 μm midi-GAGR, 6 μg/ml DMF, or 100 nm CDDO-TFEA for 3 h; fixed; and stained with antibodies to Nrf2 and βIII tubulin along with DAPI. The intensities of nuclear Nrf2 were then quantified using Metamorph and used to calculate mean ± S.E. As expected, all Nrf2 activators significantly increased the nuclear localization of Nrf2 in mouse cortical neurons compared with control: control, 14.31 ± 0.62; mini-GAGR, 34.12 ± 1.19; midi-GAGR, 38.97 ± 1.10; DMF, 29.98 ± 1.30; and CDDO-TFEA, 25.23 ± 1.76 (Fig. 3, *A* and *B*). Then we examined the extent to which a 3-h treatment increased the transcriptional activity of Nrf2 in the neurons. Intriguingly, only mini-GAGR and DMF increased the transcriptional activity of Nrf2 to statistically significant levels compared with control, whereas midi-GAGR and CDDO-TFEA did not after 3-h treatment (Fig. 3*C*). To look at the effects of the Nrf2 activators on the downstream antioxidant system, we measured the protein concentrations of two antioxidant enzymes, SOD1 and GPx4, after 48-h treatment. Mini-GAGR, midi-GAGR, DMF, and CDDO-TFEA significantly increased the protein levels of SOD1 and GPx4 compared with control (Fig. 3, *D* and *E*). The protein band densities of SOD1 and GPx4 were measured using ImageJ to calculate the average density per protein. The mean ± S.E. of SOD1 were 0.200 ± 0.047 for control, 0.427 ± 0.033 for mini-GAGR, 0.489 ± 0.030 for midi-GAGR, 0.361 ± 0.046 for DMF, and 0.525 ± 0.029 for CDDO-TFEA. The mean ± S.E. of GPx4 were 0.400 ± 0.055 for control, 0.636 ± 0.029 for mini-GAGR, 0.625 ± 0.021 for midi-GAGR, 0.604 ± 0.059 for DMF, and 0.647 ± 0.042 for CDDO-TFEA. These results suggest that mini-GAGR has an Nrf2-activating effect comparable with that of the Nrf2 activators, DMF and CDDO-TFEA, whereas midi-GAGR appears to be a slow Nrf2 activator.

### Mini-GAGR increases antioxidant enzymes and reduces free radicals under oxidative stress

**Figure 4.**
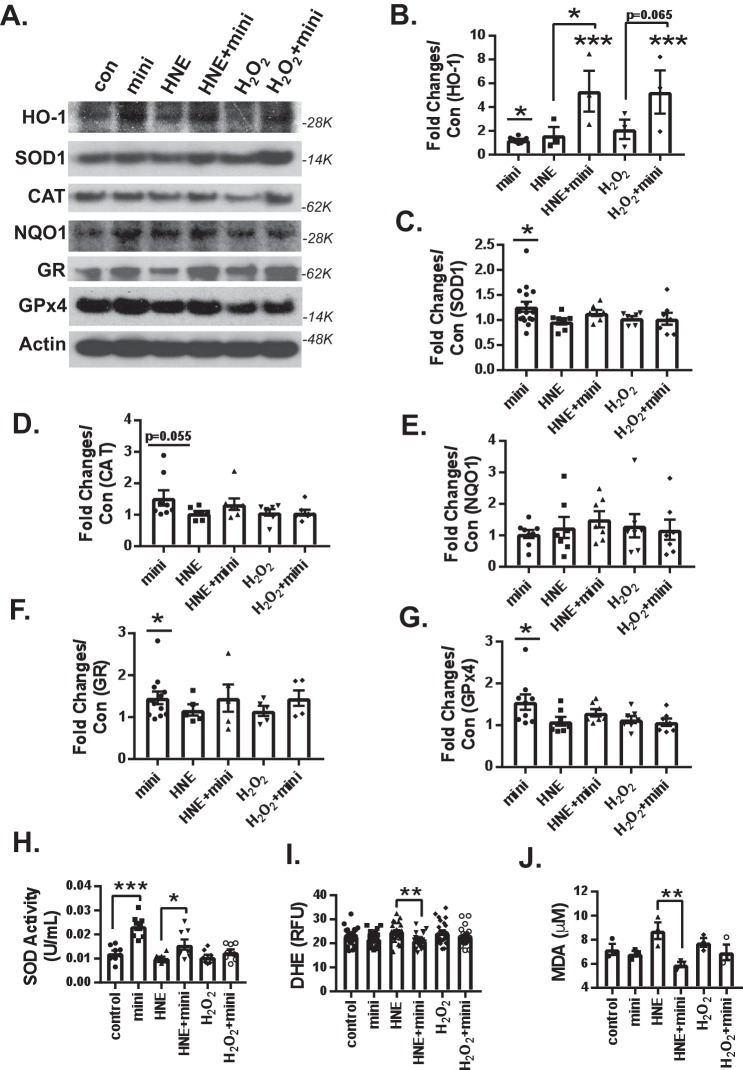
**Mini-GAGR increases the protein level of HO-1 and the enzymatic activity of SOD and reduces 4HNE-induced oxidative stress in neurons under oxidative stress.** *A*, mouse cortical neurons (E17, DIV11–14) were pretreated with either vehicle or 1 μm mini-GAGR for 16 h and treated with vehicle, 10 μm 4HNE, or 50 μm H_2_O_2_ for 30 h prior to immunoblotting, enzyme assays, and free radical measurement. The band densities of antioxidant enzyme proteins were quantified by ImageJ to obtain average density ± S.E. (*error bars*). The average density of each protein in control conditions was used to calculate -fold differences of the protein in different conditions. *B*, HO-1 (4HNE *versus* HNE + mini: *p* = 0.038, *n* = 3 different embryo batches). There were statistically significant differences in protein levels between control and mini-GAGR for HO-1 (*p* = 0.034, *n* = 7 different embryo batches (F(4, 15) = 5.1797, *p* = 0.008, ANOVA)) (*B*), SOD (*p* = 0.044, *n* = 7 different embryo batches (F(4, 39) = 1.7323, *p* = 0.162, ANOVA)) (*C*), CAT (trending, *p* = 0.055, *n* = 7 different embryo batches (F(4, 31) = 1.887, *p* = 0.138, ANOVA)) (*D*), GR (*p* = 0.022, *n* = 7 different embryo batches) (*F*), and GPx4 (*p* = 0.022, *n* = 5 different embryo batches) (*G*). There were no statistically significant differences in protein levels between free radical alone (4HNE (*HNE*) or H_2_O_2_) and free radical plus mini-GAGR for SOD (*n* = 7 different embryo batches) (*C*), CAT (*n* = 7 different embryo batches) (*D*), NQO1 (*n* = 7 different embryo batches (F(4, 31) = 0.36323, *p* = 0.833, ANOVA)) (*E*), GR (*n* = 7 different embryo batches (F(4, 27) = 0.64436, *p* = 0.636, ANOVA)) (*F*), and GPx4 (*n* = 5 different embryo batches (F(4, 32) = 2.7775, *p* = 0.044, ANOVA)) (*G*). *H*, SOD activity (units/ml) (*n* = 8 different embryo batches): control *versus* mini-GAGR (*p* < 0.001), HNE *versus* HNE + mini-GAGR (*p* = 0.028) (F(5, 42) = 13.466, *p* < 0.001, ANOVA). *I*, ROS level (HNE *versus* HNE + mini-GAGR: *p* = 0.006, *n* = 24 different embryo batches (F(5, 138) = 3.496, *p* = 0.005, ANOVA)). *J*, MDA level (HNE *versus* HNE + mini-GAGR: *p* = 0.006, *n* = 3 different embryo batches (F(5, 12) = 3.989, *p* = 0.023, ANOVA)). (*, *p* < 0.05; **, *p* < 0.01; ***, *p* < 0.001, one-way ANOVA and Bonferroni's multiple-comparison test). Data are expressed as mean ± S.E.

We speculated that mini-GAGR uses its ability to increase antioxidant enzymes and activate Nrf2 (Figs. 2 and 3) to protect neurons from oxidative stress (28). If so, mini-GAGR should be able to increase antioxidant enzymes even in the presence of free reactive radicals, such as H_2_O_2_ and 4HNE. Mouse cortical neurons (E17, DIV11–14) were pretreated for 16 h with either vehicle or 1 μm mini-GAGR and treated for 30 h with vehicle, 10 μm 4HNE, or 50 μm H_2_O_2_ before cytosol extraction from treated neurons. Mini-GAGR alone appeared to increase the protein levels of HO-1, SOD1, CAT, GR, and GPx but not NQO1 and β-actin compared with control in the absence of the free radicals (Fig. 4*A*). The band densities of the proteins were measured using ImageJ. To calculate -fold change, the protein bands in different conditions were divided by that of control conditions for each protein. HO-1 showed similar -fold changes in neurons treated with mini-GAGR, 4HNE, or H_2_O_2_ compared with control (-fold change *versus* control: 1.23 ± 0.09 for mini-GAGR, 1.63 ± 0.70 for HNE, and 2.13 ± 0.82 for H_2_O_2_) (Fig. 4*B*). Conversely, mini-GAGR significantly increased HO-1 levels in neurons that were exposed to 4HNE (5.34 ± 1.72) compared with those treated with 4HNE alone (1.63 ± 0.70). Neurons treated with mini-GAGR and H_2_O_2_ showed only a trend of increase in HO-1 level compared with those with H_2_O_2_ alone. SOD1, CAT, GR, GPx4, and NQO1 showed trends of increases in neurons treated with 4HNE after mini-GAGR pretreatment (SOD1 (Fig. 4*C*; -fold change *versus* control: 1.27 ± 0.10 for mini-GAGR, 0.97 ± 0.06 for 4HNE, and 1.14 ± 0.06 for 4HNE + mini-GAGR), CAT (Fig. 4*D*; 1.54 ± 0.24 for mini-GAGR, 1.05 ± 0.07 for 4HNE, and 1.34 ± 0.18 for 4HNE + mini-GAGR), GR (Fig. 4*F*; 1.46 ± 0.15 for mini-GAGR, 1.17 ± 0.13 for 4HNE, and 1.46 ± 0.32 4HNE + mini-GAGR), GPx4 (Fig. 4*G*; 1.56 ± 0.18 for mini-GAGR, 1.09 ± 0.11 for 4HNE, and 1.30 ± 0.09 for 4HNE + mini-GAGR), and NQO1 (Fig. 4*E*; 1.46 ± 0.15 for mini-GAGR, 1.17 ± 0.13 for 4HNE, and 1.46 ± 0.32 for 4HNE + mini-GAGR)). Conversely, pretreatment with mini-GAGR did not increase significantly SOD1, CAT, and GR in H_2_O_2_-exposed neurons (SOD1 (Fig. 4*C*; 1.04 ± 0.04 for H_2_O_2_ and 1.03 ± 0.12 for H_2_O_2_ + mini-GAGR), CAT (Fig. 4*D*; 1.08 ± 0.10 for H_2_O_2_ and 1.07 ± 0.09 for H_2_O_2_ + mini-GAGR), GR (Fig. 4*F*; 1.15 ± 0.12 for H_2_O_2_, 1.45 ± 0.18 for H_2_O_2_ + mini), and GPx4 (Fig. 4*G*; 1.14 ± 0.09 for H_2_O_2_ and 1.07 ± 0.09 for H_2_O_2_ + mini-GAGR)). NQO1 and β-actin did not show any changes in any given condition (NQO1 (Fig. 4*F*), 1.04 ± 0.13 for mini-GAGR, 1.25 ± 0.33 for 4HNE, 1.51 ± 0.26 for 4HNE + mini-GAGR, 1.30 ± 0.37 for H_2_O_2_, and 1.18 ± 0.32 for H_2_O_2_ + mini-GAGR). These results suggest 1) that mini-GAGR drastically increases HO-1 protein expression, specifically in the presence of oxidative stress; 2) that protein levels of antioxidant enzymes do not appear to be significantly decreased by 4HNE and H_2_O_2_; and 3) that mini-GAGR may increase the protein levels of SOD1, CAT, GR, GPx4, and NQO1 in mouse cortical neurons exposed to 4HNE, although to a lesser extent.

As mini-GAGR appears to affect protein expression of antioxidant enzymes under resting and oxidative stress conditions, we wondered whether mini-GAGR affected antioxidant enzymatic activity and intracellular levels of ROS and lipid radicals. First, we examined the extent to which mini-GAGR affected SOD enzymatic activity. Mouse cortical neurons (E17, DIV11–14) were pretreated for 16 h with either vehicle or 1 μm mini-GAGR and then treated with vehicle (control), 10 μm 4HNE, or 50 μm H_2_O_2_ for 30 h. Treated neurons were processed to obtain cell cytosol for measuring the enzymatic activity of SOD in the cytosols according to the manufacturer's protocol (Cayman Chemical). The measured enzymatic activities (units/ml, mean ± S.E.) of SOD were as follows: 0.012 ± 0.001 for control, 0.023 ± 0.002 for mini-GAGR, 0.010 ± 0.001 for 4HNE, 0.016 ± 0.002 for 4HNE + mini-GAGR, 0.011 ± 0.001 for H_2_O_2_, and 0.013 ± 0.001 for H_2_O_2_ + mini-GAGR. Under resting conditions, mini-GAGR increased the enzymatic activity of SOD by ∼2-fold compared with control (Fig. 4*H*). Treatment with either 4HNE or H_2_O_2_ did not significantly reduce the enzymatic activity of SOD. Conversely, pretreatment with mini-GAGR still increased that of SOD significantly in neurons exposed to 4HNE but not in those exposed to H_2_O_2_ (Fig. 4*H*). These results suggest that mini-GAGR increases the enzymatic activity of SOD under basal conditions and 4HNE.

Because mini-GAGR affects the protein expression of antioxidant enzymes under resting and potentially oxidative stress conditions, it is possible that it affects the intracellular levels of free radicals. To examine this possibility, we used a commercial dihydroethidium (DHE) kit (Cayman Chemical) and malondialdehyde (MDA) kit (Abcam) to measure intracellular levels of ROS and lipid peroxides in neurons in different conditions. Neurons were pretreated for 16 h with either vehicle or 1 μm mini-GAGR and then treated with vehicle (control), 10 μm 4HNE, or 50 μm H_2_O_2_ for 30 h prior to cytosol extraction for DHE and MDA measurement. The measured ROS levels (ROS/DHE fluorescence (RFU), mean ± S.E.) were as follows: 23.01 ± 0.73 for control, 21.95 ± 0.59 for mini-GAGR, 24.72 ± 0.77 for 4HNE, 21.26 ± 0.59 for 4HNE + mini-GAGR, 24.50 ± 0.95 for H_2_O_2_, and 22.59 ± 0.75 for H_2_O_2_ + mini-GAGR. The measured MDA levels were as follows: 7.20 ± 0.45 μm for control, 6.84 ± 0.24 μm for mini-GAGR, 8.76 ± 0.70 μm for 4HNE, 5.94 ± 0.22 μm for 4HNE + mini-GAGR, 7.78 ± 0.38 μm for H_2_O_2_, and 6.95 ± 0.65 μm for H_2_O_2_ + mini-GAGR. Mini-GAGR alone did not increase either ROS or lipid peroxide (Fig. 4, *I* and J). Exposure to 4HNE appeared to increase lipid peroxides but not ROS, whereas mini-GAGR reduced both ROS and 4HNE significantly in 4HNE-exposed neurons (Fig. 4*I*). Conversely, exposure to H_2_O_2_ did not affect the intracellular level of lipid peroxide and ROS, and mini-GAGR did not decrease lipid peroxide and ROS significantly in H_2_O_2_-exposed neurons (Fig. 4, *I* and *J*). Thus, mini-GAGR appears to be able to reduce the intracellular levels of ROS and lipid peroxide in neurons exposed to specifically 4HNE.

### Mini-GAGR protects mitochondria from oxidative insults

In AD brain, the mitochondria is the most susceptible to breaking down because it is easily damaged by oxidative stress (1). Therefore, reducing oxidative stress should protect mitochondria from oxidative damage. We examined the ability of mini-GAGR to protect mitochondria from oxidative stress using MitoTracker and JC-1 staining.

**Figure 5.**
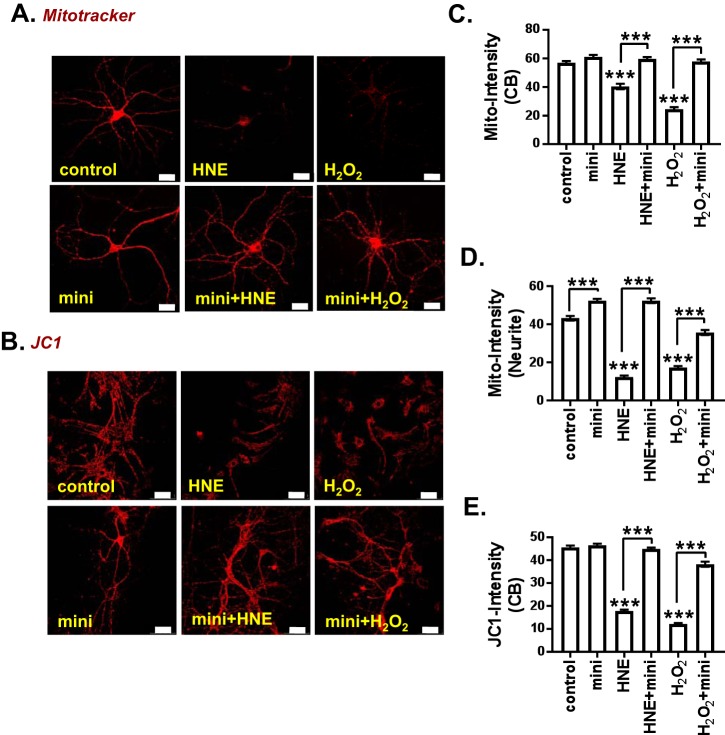
**Mini-GAGR protects mitochondria and MMP from oxidative stress.** *A*, mouse cortical neurons (E17, DIV11–14) were pretreated with either vehicle (control) or 1 μm mini-GAGR (*mini*) for 16 h and treated with vehicle, 10 μm 4HNE (*HNE*), or 50 μm H_2_O_2_ for 30 h prior to MitoTracker (*A*) or JC-1 (*B*) staining. The intensities of MitoTracker in the cell body (*CB*) (*C*) and neurites (*D*) of fixed neurons were quantified by Metamorph software. *C*, average intensity ± S.E. (*error bars*) of MitoTracker staining in cell body (*n* = 100 cell bodies): control *versus* HNE (*p* < 0.001), control *versus* H_2_O_2_ (*p* < 0.001), HNE *versus* HNE + mini-GAGR (*p* < 0.001), H_2_O_2_ *versus* H_2_O_2_ + mini-GAGR (*p* < 0.001) (F(5, 638) = 115.97; *p* < 0.001, ANOVA). *D*, average intensity ± S.E. of MitoTracker in neurites (*n* = 120 neurites): control *versus* mini-GAGR (*p* < 0.001), control *versus* HNE (*p* < 0.001), control *versus* H_2_O_2_ (*p* < 0.001), HNE *versus* HNE + mini-GAGR (*p* < 0.001), H_2_O_2_ *versus* H_2_O_2_ + mini-GAGR (*p* < 0.001) (F(5, 746) = 241.14; *p* < 0.001, ANOVA). *E*, JC-1 (*n* = 200 cell bodies): control *versus* HNE (*p* < 0.001), control *versus* H_2_O_2_ (*p* < 0.001), HNE *versus* HNE + mini-GAGR (*p* < 0.001), H_2_O_2_ *versus* H_2_O_2_ + mini-GAGR (*p* < 0.001) (F(5, 1428) = 445.69; *p* < 0.001, ANOVA). ***, *p* < 0.001, one-way ANOVA and Bonferroni's multiple-comparison test; *scale bar*, 30 μm). Data are expressed as mean ± S.E. (Of note, because the total number of measurements per condition was over 100, scatter plots were not used for bar graphs.)

MitoTracker is used to detect the membrane integrity of mitochondria. Mouse cortical neurons (E17, DIV11–14) were pretreated for 16 h with either vehicle or 1 μm mini-GAGR and then treated with vehicle, 10 μm 4HNE, or 50 μm H_2_O_2_ for 30 h prior to MitoTracker staining (0.3 μm) at 37 °C for 30 min. After fixation in 3.7% paraformaldehyde, neurons were imaged by confocal microscopy. The intensities of MitoTracker staining along the proximal neurites (100 μm from cell body, 1–2 neurites/cell body) and in the cell bodies of neurons were analyzed using Metamorph software. In cell bodies, mini-GAGR treatment slightly increased average intensity in cell bodies (mean ± S.E.; 61.10 ± 1.38, *n* = 115) compared with vehicle treatment (59.96 ± 1.20, *n* = 103) (Fig. 5*C*). Treatments with 4HNE and H_2_O_2_ decreased MitoTracker staining in cell bodies significantly to 40.38 ± 1.89 (*n* = 92) and 24.46 ± 1.39 (*n* = 137), respectively (*p* < 0.001). On the other hand, pretreatment of mini-GAGR restored the intensity of MitoTracker staining in the cell bodies of the neurons treated with 4HNE and H_2_O_2_ to the control level (59.62 ± 1.35 (*n* = 111) for 4HNE + mini-GAGR and 57.84 ± 1.52 (*n* = 86) for H_2_O_2_ + mini-GAGR) (Fig. 5*C*). In proximal neurites, mini-GAGR treatment increased the average intensity of MitoTracker (52.40 ± 1.02, *n* = 126) compared with vehicle treatment (43.22 ± 1.18, *n* = 118) (*p* < 0.001) (Fig. 5*D*). 4HNE and H_2_O_2_ decreased MitoTracker staining in proximal neurites drastically to 12.34 ± 0.77 (*n* = 117) and 17.31 ± 0.82 (*n* = 122), respectively (*p* < 0.001). On the other hand, mini-GAGR treatment restored the intensity of MitoTracker in the proximal neurites of neurons treated with 4HNE to the level of control (52.37 ± 1.25 (*n* = 128)) (*p* < 0.001) (Fig. 5*D*). Mini-GAGR treatment also reversed the loss of the intensity of MitoTracker in the proximal neurites of neurons treated with H_2_O_2_ to 35.70 ± 1.31 (*n* = 141) (Fig. 5*D*). Thus, it is clear that mini-GAGR treatment protects mitochondrial membrane integrity in the cell body from oxidative stress caused by 4HNE and H_2_O_2_ analogous to control level. In the proximal neurites, mini-GAGR treatment protects mitochondria from 4HNE comparably with the control level, whereas protection from H_2_O_2_ was about ∼68% restoration of the control level.

JC-1 is a marker of MMP. At low concentrations (low MMP), JC-1 is predominantly a monomer that yields green fluorescence (530 ± 15 nm). At high concentrations (high MMP), JC-1 aggregates inside mitochondria, yielding a red-to-orange-colored emission (590 ± 17.5 nm) (47). Mouse cortical neurons (E17, DIV11–14) cultured in a live cell chamber were treated as above and stained with 2 μm JC-1 at 37 °C for 30 min. Immediately after staining, the red fluorescence of JC-1 was captured by confocal microscopy, and intensities in cell bodies were analyzed using Metamorph software. Compared with those treated with either 4HNE or H_2_O_2_ alone, mini-GAGR significantly increased JC-1 intensity in the cell bodies of neurons treated with either free radical (Fig. 5*E*). The intensities (mean ± S.E.) of JC-1 are as follows: control (45.61 ± 0.82) (*n* = 189), mini-GAGR (46.52 ± 0.72) (*n* = 281), 4HNE (17.80 ± 0.57) (*n* = 278), 4HNE + mini-GAGR (44.93 ± 0.57) (*n* = 284), H_2_O_2_ (12.16 ± 0.40) (*n* = 193), and H_2_O_2_ + mini-GAGR (38.27 ± 1.09) (*n* = 209) (Fig. 5*E*). Although we also imaged the green fluorescence of JC-1, the intensities were not analyzed because of the diffusion of green fluorescent JC-1 throughout the cytoplasm and its inconsistent staining among neurons on the same coverslip under the same conditions.

### Nrf2 knockdown reduces mini-GAGR–induced increase in antioxidant enzyme proteins

To know whether Nrf2 is required for mini-GAGR–mediated increases in antioxidant enzyme proteins, we knocked down Nrf2 using Nrf2 shRNA (anti-mouse Nrf2) lentiviral particles along with control shRNA. For an unknown reason, both shRNAs caused a reduction in overall protein levels in transfected neurons, implying that the shRNAs may weaken neurons. Thus, we normalized the band densities of each protein per condition using that of actin for the same condition and then calculated the average of normalized protein band densities for bar graphs. The protein level of Nrf2 in vehicle-treated neurons was reduced by 30% by anti-Nrf2 shRNA (protein band density (mean ± S.E.): 0.59 ± 0.02 for control shRNA *versus* 0.41 ± 0.01 for Nrf2 shRNA, *p* < 0.01) (Fig. S1, *A* and *B*). Mini-GAGR treatment did not increase Nrf2 in neurons transfected with either control shRNA (0.62 ± 0.03) or Nrf2 shRNA (0.45 ± 0.01) (Fig. S1, *A* and *B*). In control shRNA–transfected neurons, mini-GAGR increased protein levels of HO-1 and GPx4 (control *versus* mini-GAGR: 0.32 ± 0.03 *versus* 0.46 ± 0.01 (*p* < 0.05) for HO-1 (Fig. S1, *A* and *C*) and 0.76 ± 0.05 *versus* 1.03 ± 0.08 (*p* < 0.05) for GPx4 (Fig. S1, *A* and *G*). In control shRNA–transfected neurons, however, mini-GAGR did not significantly increase SOD1, NQO1, CAT, and GR (control *versus* mini-GAGR: 0.95 ± 0.02 *versus* 0.93 ± 0.02 for SOD1 (Fig. S1, *A* and *D*), 0.25 ± 0.05 *versus* 0.28 ± 0.02 for NQO1 (data not shown), 0.29 ± 0.05 *versus* 0.40 ± 0.04 for CAT (*p* < 0.01) (Fig. S1, *A* and *E*), and 0.40 ± 0.02 *versus* 0.48 ± 0.06 for GR (Fig. S1, *A* and *F*)). On the other hand, in Nrf2 shRNA–transfected neurons, mini-GAGR could not increase any of the antioxidant enzymes (control *versus* mini-GAGR: 0.35 ± 0.04 *versus* 0.36 ± 0.02 for HO-1 (Fig. S1*C*), 0.16 ± 0.05 *versus* 0.11 ± 0.06 for CAT (Fig. S1*E*), 0.47 ± 0.15 *versus* 0.33 ± 0.02 for GPx4 (Fig. S1*G*), 0.88 ± 0.05 *versus* 0.79 ± 0.01 for SOD1 (Fig. S1*D*), 0.29 ± 0.01 *versus* 0.17 ± 0.02 for NQO1 (data not shown), and 0.35 ± 0.03 *versus* 0.17 ± 0.01 for GR (Fig. S1*F*)). These results suggest that the 30% reduction of Nrf2 blocks mini-GAGR–induced increase in the protein levels of HO-1 and GPx4.

### Mini-GAGR decreases Nrf2 and Keap1 interaction

**Figure 6.**
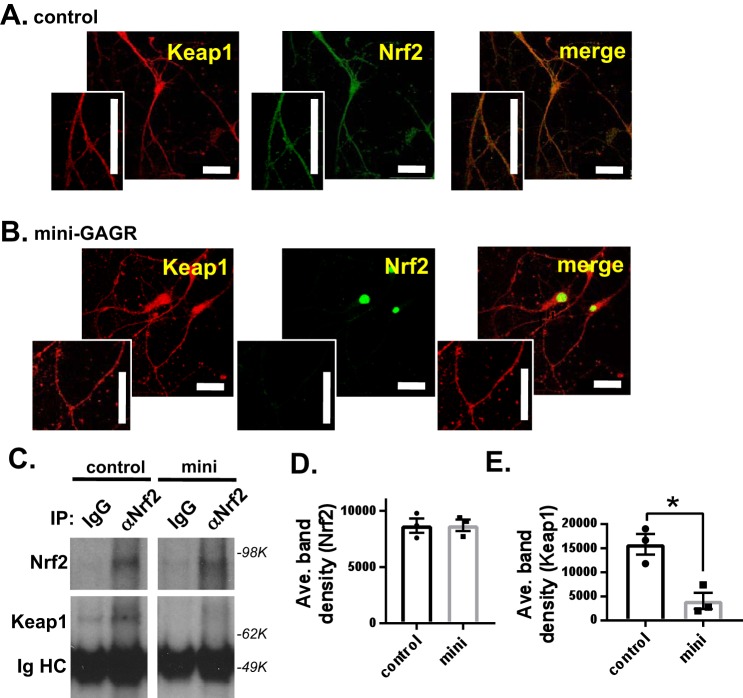
**Mini-GAGR reduces the colocalization and interaction between Keap1 and Nrf2.** *A* and *B*, mouse cortical neurons (E17, DIV 14) were treated with 1 μm mini-GAGR (*mini*) for 0 h (*A*) and 3 h (*B*) and processed for immunocytochemistry with primary antibodies against Nrf2 (*green*) and Keap1 (*red*) and secondary antibodies (Alexa Fluor 488 and Alexa Fluor 594), Pearson's colocalization coefficient *R* was quantified using ImageJ. *Scale bar*, 30 μm. *C*, mouse cortical neurons (E17, DIV 14) were treated with either vehicle (control) or 1 μm mini-GAGR (*mini*) for 3 h and lysed to obtain cytosols for co-IP using either control IgG or anti-Nrf2 IgG (α*Nrf2*). The band densities of precipitated Nrf2 (80 kDa) and Keap1 (70 kDa) proteins were quantified by ImageJ to obtain average density ± S.E. (*error bars*) for the bar graphs. *D*, Nrf2, control *versus* mini-GAGR. *E*, Keap1, control *versus* mini-GAGR (*n* = 3 different embryo batches; *, *p* < 0.05; Student's *t* test, two-tailed). Data are expressed as mean ± S.E.

It is thought that Nrf2 is activated by its dissociation from its repressor protein, Keap1 (14). Thus, it is possible that mini-GAGR acts by causing the dissociation of Nrf2 from Keap1. First, we examined the colocalization between Nrf2 and Keap1 in neurons before and after mini-GAGR treatment. Mouse cortical neurons (E17, DIV13) were treated with 1 μm mini-GAGR for 0 or 3 h, fixed, and stained with antibodies to Keap1 and Nrf2. At 0 h, Nrf2 resided with Keap1 along the neurites and cell body (Fig. 6*A*). At 3 h, most Nrf2 was translocated into the nuclei and still colocalized with Nrf2 in the cell body (Fig. 6*B*). We used the colocalization program JACoP of ImageJ to measure Pearson's coefficient *R* of the colocalization between Nrf2 and Keap1 in those neurons. At 0 h, Pearson's coefficient *R* was 0.69 ± 0.01, which indicates significant colocalization between them. Pearson's coefficient *R* was decreased to 0.57 ± 0.01 after a 3-h treatment with mini-GAGR. The decrease in Pearson's coefficient *R* appears to be due to the reduction of Nrf2 along the neurites. This result suggests that Nrf2 in the cytoplasm loses its colocalization with Keap1 after mini-GAGR treatment. Then we performed co-immunoprecipitation (co-IP) using anti-Nrf2 rabbit antibody (rabbit IgG as control). Neurons treated with either vehicle (control) or 1 μm mini-GAGR for 3 h were processed to obtain cytosol for co-IP. The protein levels of Keap1 in immunoprecipitated proteins were detected by anti-Keap1 mouse antibody, and the densities of their protein bands were measured using ImageJ. Anti-Nrf2 antibody precipitated a similar amount of Nrf2 from the cytosols of the neurons treated with either vehicle or mini-GAGR (band density: 8696.7 ± 633.6 for control *versus* 8718.9 ± 514.7 for mini-GAGR) (Fig. 6, *C* and *D*). Conversely, the amount of Keap1 co-precipitated with Nrf2 was significantly decreased (∼74%) by mini-GAGR treatment compared with that by vehicle treatment (band density: 15,829.3 ± 2123.9 for control *versus* 4072.5 ± 1679.3 for mini-GAGR, *p* < 0.01) (Fig. 6, *C* and *E*). Thus, it is clear that mini-GAGR causes the dissociation of Nrf2 from Keap1 in mouse cortical neurons.

### Mini-GAGR induces Nrf2 phosphorylation and nuclear translocation in a PKC- and FGFR1-dependent manner

**Figure 7.**
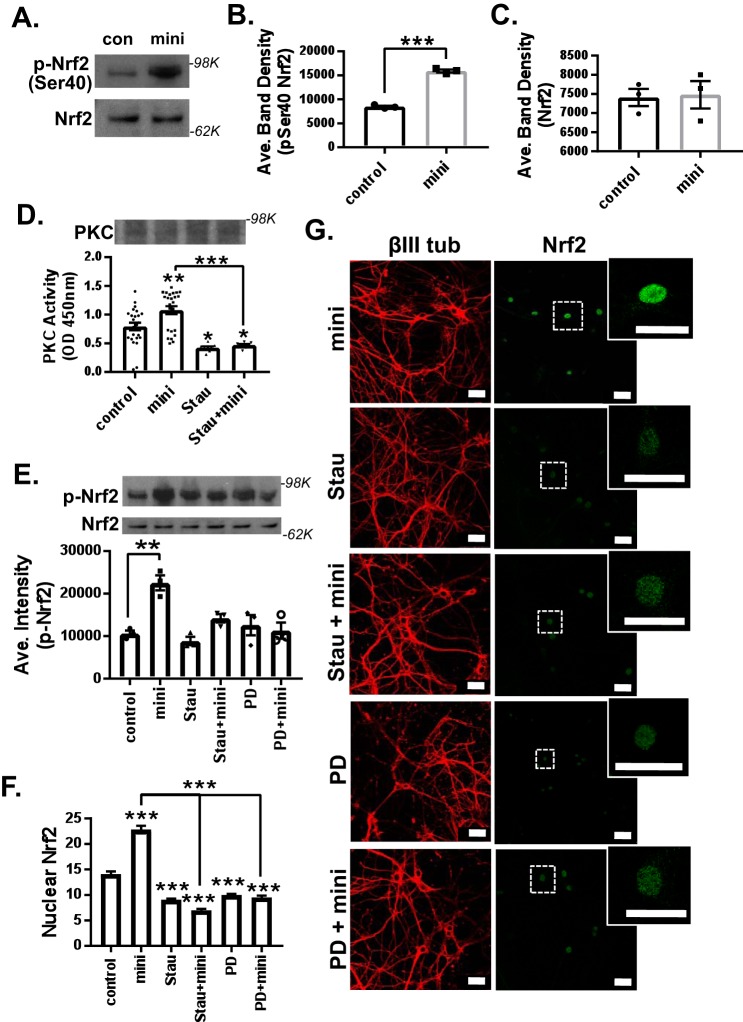
**Mini-GAGR induces phosphorylation and nuclear localization of Nrf2 in a PKC- and FGFR1-dependent manner.** *A*, mouse cortical neurons (E17, DIV14) were treated with 1 μm mini-GAGR (*mini*) for 3 h and processed for immunoblotting (p-Ser-40-Nrf2 and Nrf2). The band densities of phospho-Nrf2 (100 kDa) (*B*) and total Nrf2 (80 kDa) (*C*) were quantified by ImageJ to obtain average density ± S.E. (*error bars*) of p-Nrf2 (*p* < 0.001, *n* = 3 different embryo batches), Nrf2 protein concentration (*n* = 3 different embryo batches) (Student's *t* test, two-tailed). *D*, primary cortical neurons (E17, DIV14) were pretreated with either vehicle or 3 nm Stau (PKC inhibitor) for 2 h and treated with either vehicle (control) or 1 μm mini-GAGR for 3 h prior to the measurement of PKC activity (*n* = 8 different embryo batches): control *versus* mini-GAGR (*mini*) (*p* = 0.008), control *versus* Stau (*p* = 0.017), control *versus* Stau + mini-GAGR (*p* = 0.047), mini-GAGR *versus* Stau + mini-GAGR (*p* < 0.001) (one-way ANOVA (F(3, 60) = 14.631, *p* < 0.001, ANOVA, and Bonferroni's multiple-comparison test). *E–G*, primary cortical neurons (E17, DIV14) were pretreated with vehicle, 3 nm staurosporine, or 50 nm PD173074 (FGFR1 inhibitor) for 2 h and treated with 1 μm mini-GAGR for 3 h prior to immunoblotting (p-Nrf2 and Nrf2). *E*, the band densities of antioxidant enzyme proteins were quantified by ImageJ to obtain average density ± S.E. for bar graphs: control *versus* mini-GAGR (*n* = 4 different embryo batches, *p* < 0.01 (F (5, 12) = 9.4859, *p* < 0.001, ANOVA)). *F* and *G*, primary cortical neurons (E17, DIV14) were pretreated with vehicle, 3 nm staurosporine, or 50 nm PD173074 for 2 h and treated with 1 μm mini-GAGR for 3 h prior to immunocytochemistry (Nrf2 (*green*; Alexa Fluor 488), βIII-tubulin (*red*; Alexa Fluor 594), DAPI). *F*, the fluorescence intensities of nuclear Nrf2 in images were quantified using Metamorph to calculate the average intensity ± S.E. of nuclear Nrf2 staining for bar graphs (*n* = 100–123 cells): control *versus* mini-GAGR, Stau, Stau + mini-GAGR, PD, PD + mini-GAGR (*p* < 0.001); mini-GAGR *versus* Stau + mini-GAGR, PD + mini-GAGR (*p* < 0.001) (F(5, 877) = 137.67, *p* < 0.001, ANOVA). *, *p* < 0.05; **, *p* < 0.01; ***, *p* < 0.001, one-way ANOVA and Bonferroni's multiple-comparison test). Data are expressed as mean ± S.E. (*scale bar*, 30 μm). (Of note, because the total number of measurements per condition was over 100, scatter plots were not used for graphs.)

Because the Ser-40 phosphorylation of Nrf2 is known to activate Nrf2 and induce its translocation into the nucleus (48), we examined the ability of mini-GAGR to increase Nrf2 Ser-40 phosphorylation in mouse cortical neurons. Mouse cortical neurons (E17, DIV13) were treated with either vehicle or 1 μm mini-GAGR for 3 h and processed for immunoblotting. 3-h treatment with mini-GAGR significantly increased Ser-40 phosphorylation of Nrf2 (band density: 8475.74 ± 215.28 for control *versus* 15,922.96 ± 314.33 for mini-GAGR, *p* < 0.001) without changing the total amounts of Nrf2 (band density: 7406.73 ± 224.89 for control *versus* 7478.53 ± 358.45 for mini-GAGR) (Fig. 7, *A–C*). Given that PKC mediates the Ser-40 phosphorylation of Nrf2 (48), it is possible that mini-GAGR treatment activates PKC. Thus, we examined the kinase activity (*A*_450_) of PKC in neurons treated with either vehicle or 1 μm mini-GAGR for 2 h using a commercial kit (Abcam). Neurons were also pretreated with PKC inhibitor, staurosporine (Stau; 3 nm), prior to treatment with vehicle or mini-GAGR to specify any kinase activity to PKC. As a result, mini-GAGR treatment significantly increased PKC activity compared with control cells, and staurosporine blocked mini-GAGR–induced PKC activity (Fig. 7*D*). The protein levels of PKC were not affected by any conditions (Fig. 7*D*). The observed PKC activities (*A*_450_) in different treatments are as follows: control (0.79 ± 0.07), mini-GAGR (1.08 ± 0.07), Stau (0.43 ± 0.02), and Stau + mini-GAGR (0.47 ± 0.02). Thus, it is clear that mini-GAGR activates PKC. Next, we examined whether staurosporine blocks mini-GAGR–induced Ser-40 phosphorylation of Nrf2 or not by pretreating neurons with 3 nm staurosporine (2 h) prior to treatment with vehicle or mini-GAGR. As expected, staurosporine blocked the Ser-40 phosphorylation of Nrf2 in mini-GAGR-treated neurons (Fig. 7*E*). We also examined whether FGFR1 that interacts with midi-GAGR (28) is involved in mini-GAGR–mediated Nrf2 phosphorylation by pretreating neurons with 50 nm PD173074 (PD; 2 h), an FGFR1 inhibitor, prior to the treatment with either vehicle or mini-GAGR. The FGFR1 inhibitor also blocked Ser-40 phosphorylation of Nrf2 in mini-GAGR–treated neurons (Fig. 7*E*). This result indicates that FGFR1 is also involved in mini-GAGR–mediated Nrf2 phosphorylation. The band densities of p-Nrf2 are as follows: 10,596 ± 700.3 for control, 15,922.96 ± 314.33 for mini-GAGR, 8844 ± 985.6 for Stau, 14,203 ± 982.5 for Stau + mini-GAGR, 12,593 ± 2399.2 for PD, and 11,305 ± 1901.6 for PD + mini-GAGR. There was no significant difference in the total amounts of Nrf2 in these different inhibitor conditions. Finally, we examined whether PKC and FGFR1 are involved in mini-GAGR–induced translocation of Nrf2 into the nucleus. Mouse cortical neurons (E17, DIV13) were pretreated with either 3 nm staurosporine or 50 nm PD173074 for 3 h prior to the treatment with either vehicle or 1 μm mini-GAGR for 3 h. Then neurons were fixed and stained with antibodies to βIII tubulin, Nrf2, and DAPI for confocal imaging. Both staurosporine and PD173074 reduced mini-GAGR–induced nuclear localization of Nrf2, suggesting that PKC and FGFR1 are involved in mini-GAGR–induced nuclear localization of Nrf2. The average intensities of nuclear Nrf2 in different conditions are as follows: 14.08 ± 0.50 for control, 22.82 ± 0.75 for mini-GAGR, 9.07 ± 0.19 for Stau, 6.99 ± 0.27 for Stau + mini-GAGR, 9.98 ± 0.23 for PD, and 9.51 ± 0.37 for PD + mini-GAGR. These results suggest that mini-GAGR activates PKC and FGFR1 that, in turn, phosphorylate Nrf2, causing the nuclear translocation of Nrf2 in mouse cortical neurons.

### Mini-GAGR bypasses BBB, activates Nrf2, and increases antioxidant enzymes in the hippocampus and cortex of 3xTg-AD mice

**Figure 8.**
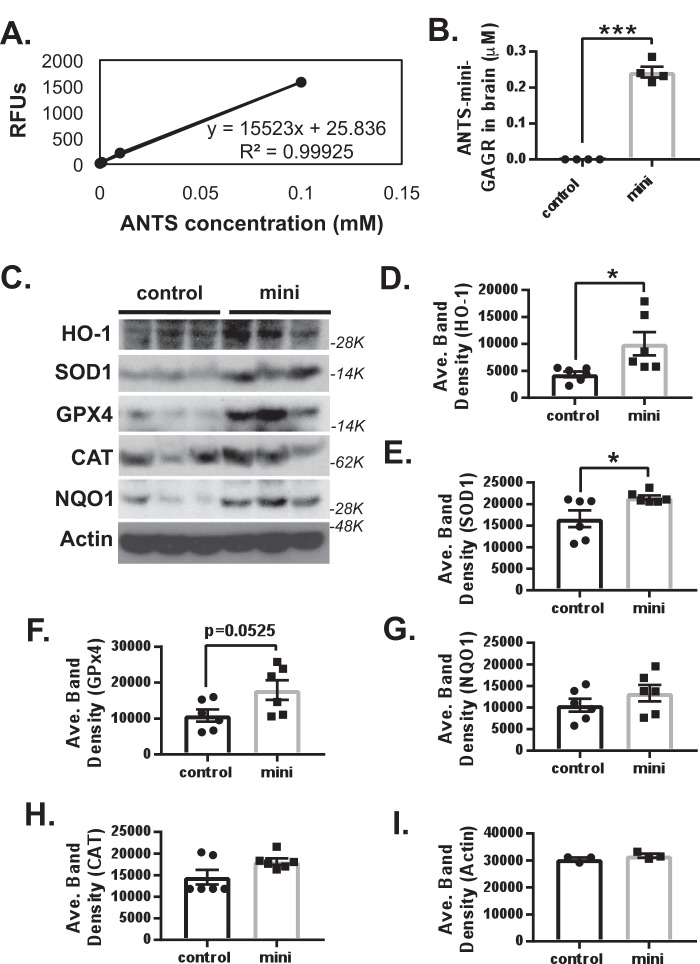
**Mini-GAGR bypasses BBB and increases HO-1, SOD1, and, maybe, GPx4 in the brains of 12-month-old female 3xTg-AD mice.** *A*, the standard curve for ANTS in brain cytosols that is used to convert the fluorescence values of ANTS to the concentrations of mini-GAGR (ANTS/mini-GAGR = 1:10) to which ANTS was conjugated. *B*, 100 nmol/40 μl ANTS-tagged mini-GAGR or vehicle (water) was administered into the nostrils of 10-month-old WT mice (average weight ∼25 g, *n* = 4 animals/treatment group). The brain cytosol was extracted from the brains of the mice and used to quantify ANTS–mini-GAGR for bar graphs (average concentration ± S.E. (*error bars*) of ANTS–mini-GAGR *versus* vehicle; ***, *p* < 0.001). *C*, 3xTg-AD mice were intranasally administered vehicle (control) or 100 nmol of mini-GAGR for 20 days (*n* = 6 animals/treatment group) and processed to obtain the cytosols of combined hippocampal and cortical tissues for immunoblotting. The band densities of antioxidant enzymes in the cytosols were quantified by ImageJ to obtain average density ± S.E. for bar graphs. *D*, HO-1 (32 kDa): control *versus* mini-GAGR (*, *p* < 0.05); *E*, SOD1 (16 kDa): control *versus* mini-GAGR (*, *p* < 0.05); *F*, GPx4 (21 kDa): control *versus* mini-GAGR (*, *p* = 0.525); *G*, NQO1 (31 kDa): control *versus* mini-GAGR; *H*, CAT (64 kDa): control *versus* mini-GAGR; *I*, β-actin (45 kDa): control *versus* mini-GAGR. (*, *p* < 0.05; ***, *p* < 0.001; unpaired *t* test, two-tailed). Data are expressed as mean ± S.E.

In our previous studies, midi-GAGR, the larger (4.7-kDa) cleavage product of low-acyl gellan gum, could bypass the BBB within 6 h (29). Therefore, it is possible that mini-GAGR (0.7 kDa), the smaller cleavage product of low-acyl gellan gum, also bypasses the BBB. To examine this possibility, we tagged mini-GAGR with the fluorescent tag, ANTS, according to our previous study (29). 100 nmol/40 μl ANTS-tagged mini-GAGR was administered into the nostrils of 10-month-old WT mice (average weight ∼25 g, *n* = 4). As a control, four WT mice were given sterile water intranasally. Mice were euthanized 6 h after the intranasal administration of ANTS–mini-GAGR for brain extraction. The whole brains of the mice were homogenized in 1 ml of milliQ water using a pestle and a 2-ml cylinder and spun for 20 min at 13,000 × *g* and for 30 min at 100,000 × *g*. The brains of water-administered mice were used as blank control. Then the supernatant (whole-brain cytosol) was used to measure the fluorescence (excitation, 350 nm; emission, 520 nm) of ANTS using a SpectraMax M5 plate reader and SoftMax Pro version 5.2 software. We generated the standard curve for ANTS in brain cytosols to convert the fluorescence values of ANTS to the concentrations of mini-GAGR (ANTS/mini-GAGR = 1:10) to which ANTS was conjugated (Fig. 8*A*). We found that ∼0.2 μm ANTS–mini-GAGR was accumulated inside the brains of the WT and 3xTg-AD mice 6 h after intranasal administration (Fig. 8*B*), suggesting that mini-GAGR is able to bypass the BBB and enter the brain.

Given that mini-GAGR can enter the brain, it is expected to affect the protein levels of antioxidant enzymes as it does in cultured mouse cortical neurons. First, we compared the protein levels of HO-1, SOD1, GPx4, CAT, NQO1, and actin in the combined tissues of hippocampus and cortex between 12-month-old WT mice (*n* = 6) and 12-month-old 3xTg-AD mice (*n* = 6) (Fig. S2). HO-1 and CAT showed statistically significant decreases in protein levels in 3xTg-AD mice compared with WT (band density: HO-1, 6756.4 ± 681.5 for WT *versus* 5024.7 ± 311.5 for 3xTg-AD mice, *p* < 0.05; CAT, 25,388.8 ± 334.4 for WT *versus* 17,900.2 ± 1379.4 for 3xTg-AD mice, *p* < 0.001). SOD1 and GPx4 showed a trend of decrease in protein levels in 3xTg-AD mice compared with WT (SOD1, 14,800.9 ± 1070.9 for WT *versus* 12,233.9 ± 625.3 for 3xTg-AD mice, *p* = 0.065; GPx4, 23,992.3 ± 833.4 for WT *versus* 19,172.9 ± 2230.5 for 3xTg-AD mice, *p* = 0.071). NQO1 and β-actin did not show any decrease compared with WT (NQO1, 9548.1 ± 493.3 for WT *versus* 10,562.7 ± 879.5 for 3xTg-AD mice; β-actin, 22,392.8 ± 617.4 for WT *versus* 22,746.8 ± 742.5 for 3xTg-AD mice). Thus, it appears that 3xTg-AD mice have lower levels of antioxidant enzymes in the hippocampus and its nearby cortex at 12 months of age compared with age-matched WT, suggesting that these decreases, significant and trending, are transgenic-dependent.

Then we examined whether mini-GAGR affects the protein levels of antioxidant enzymes in the combined tissues of the hippocampus and cortex of 3xTg-AD mice. We administered 100 nmol/40 μl mini-GAGR (20 μl/nostril) per 3xTg-AD mouse (average weight ∼25.5 g) intranasally once per day for 20 days. 40 μl of vehicle (sterile water) was also administered intranasally to 3xTg-AD mice as control for 20 days. After 14-day treatment, 3xTg-AD mice were tested for open field and Barnes maze for 5 days prior to the euthanization on the 20th day to obtain brains for either protein extraction for immunoblot or fixation for immunocytochemistry. The intranasal administration of mini-GAGR and vehicle was continued during behavior tests until euthanization. We dissected out the hippocampus and nearby cortex from 3xTg-AD mice treated with either vehicle or mini-GAGR for protein extraction for immunoblotting (*n* = 6/treatment group). Protein band densities were measured using ImageJ and used to calculate average density. We examined HO-1, SOD1, GPx4, NQO1, CAT, and β-actin (loading control). Both HO-1 and SOD1 were significantly increased in 3xTg-AD mice treated with mini-GAGR compared with those with vehicle (Fig. 8, *C–E*) (band density: HO-1, 4358.8 ± 538.7 for vehicle *versus* 10,020.3 ± 2154.3 for mini-GAGR; SOD1, 16,596.53 ± 1955.6 for vehicle *versus* 21,511.5 ± 521.9 for mini-GAGR). GPx4 showed a trend of increase in 3xTg-AD treated with mini-GAGR compared with those with vehicle (Fig. 8, *C* and *F*) (9715.6 ± 1980.7 for vehicle *versus* 16,036.1 ± 3105.6 for mini-GAGR). Conversely, NQO1, CAT, and β-actin did not show any statistically significant change in those with mini-GAGR compared with those with vehicle (Fig. 8, *C*, *G*, *H*, and *I*) (NQO1, 9418.6 ± 1507.7 for vehicle *versus* 11,923.0 ± 2159.6 for mini-GAGR; CAT, 13,043.5 ± 2105.4 for vehicle *versus* 15,988.5 ± 2447.0 for mini-GAGR; β-actin, 30,489.5 ± 650.9 for vehicle *versus* 31,784.2 ± 766.2 for mini-GAGR). Thus, 20-day intranasal treatment with mini-GAGR significantly increases the protein levels of HO-1 and SOD1 and slightly increases the level of GPx4 while not affecting the levels of NQO1, CAT, and β-actin in the hippocampus and its nearby cortex.

### Mini-GAGR decreases Nrf2 and Keap1 interaction and increases nuclear p-Nrf2 in hippocampi in 3xTg-AD brains

**Figure 9.**
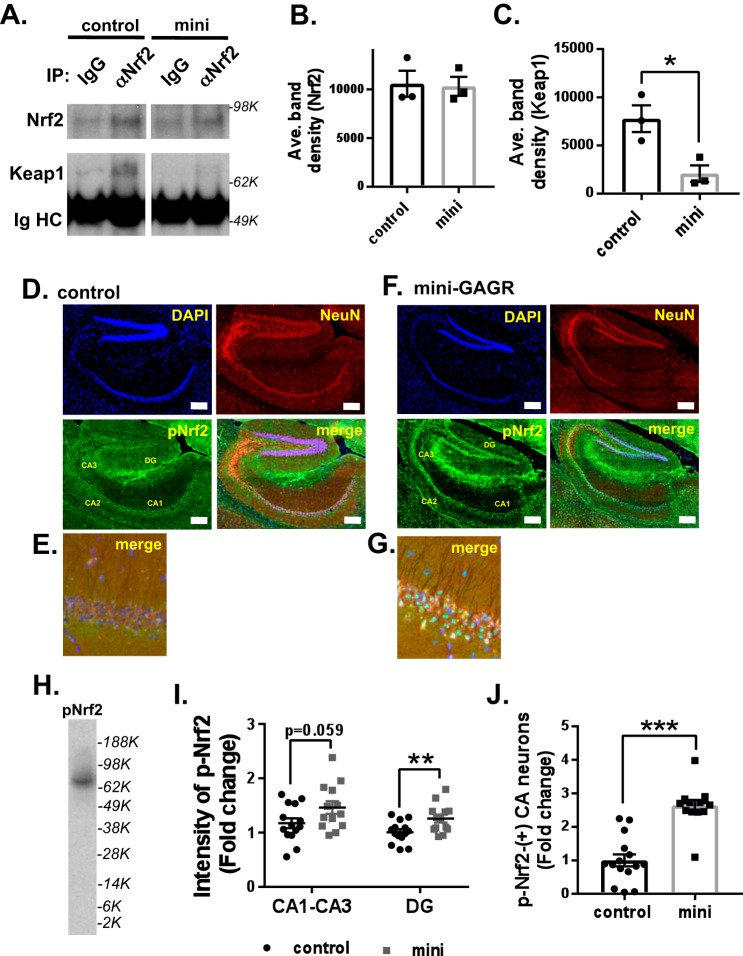
**Mini-GAGR reduces the interaction between Keap1 and Nrf2 and increases p-Nrf2 in hippocampal neurons in 12-month-old female 3xTg-AD mouse brains.** *A*, the brain cortex cytosols from 3xTg-AD mice treated with either vehicle or 100 nmol of mini-GAGR for 20 days were used for co-IP using control IgG or anti-Nrf2 IgG. Precipitated Nrf2 and Keap1 were detected by immunoblotting. The band densities of precipitated Nrf2 and Keap1 were quantified by ImageJ to obtain average density ± S.E. (*error bars*) for bar graphs. *B*, Nrf2 (80 kDa): control *versus* mini-GAGR. *C*, Keap1 (70 kDa): control *versus* mini-GAGR (*n* = 3 animals/treatment; *, *p* < 0.05). *D* and *F*, the hippocampal sections of 3xTg-AD mice treated with either vehicle (*D*) or 100 nm mini-GAGR (*F*) were stained with antibodies to NeuN (*red*; Alexa Fluor 594), and p-Nrf2 (*green*; Alexa Fluor 488) along with DAPI (*blue*) (*scale bar*, 250 μm). *E* and *G*, magnified images of the immunostained hippocampal CA1 regions of 3xTg-AD mice treated with either vehicle (*E*) or 100 nm mini-GAGR (*G*) (*scale bar*, 50 μm). *H*, mouse cortical neurons treated with 1 μm for 3 h were used to confirm the specificity of anti-p-Nrf2 antibody. *I*, bar graphs show the -fold changes of intensities of p-Nrf2 staining in hippocampal CA1-CA3 and DG regions of 3xTg-AD mice treated with either vehicle or 100 nm mini-GAGR (*n* = 6 animals (14 sections)/treatment, *p* = 0.059; **, *p* < 0.01). *J*, bar graphs show the -fold changes of p-Nrf2–positive hippocampal CA1–CA3 and DG regions of 3xTg-AD mice treated with either vehicle or 100 nm mini-GAGR (*n* = 6 animals (14 sections)/treatment; ***, *p* < 0.001). *, *p* < 0.05; **, *p* < 0.01; unpaired *t* test, two tailed). Data are expressed as mean ± S.E.

Given that intranasally administered mini-GAGR increased some antioxidant enzymes (HO-1 and SOD1) in 3xTg-AD mouse hippocampi, it is possible that it activates Nrf2 there. First, we examined the interaction between Nrf2 and Keap1 in the brains of mini-GAGR–treated 3xTg-AD mice. The brains of 3xTg-AD mice intranasally treated with either vehicle or 100 nmol/day mini-GAGR for 20 days were processed for cytosol extraction in PMEE buffer containing 1% Igepal CA-630 and the inhibitors of proteases and phosphatases. Then we performed co-IP using anti-Nrf2 rabbit antibody (rabbit IgG as control). The protein levels of Keap1 in immunoprecipitated proteins were detected by anti-Keap1 mouse antibody, and the densities of their protein bands were measured using ImageJ. Anti-Nrf2 antibody precipitated a similar amount of Nrf2 from the brain cytosols of 3xTg-AD mice treated with either vehicle (control) or mini-GAGR (band density, 10,569.9 ± 1342 for control *versus* 10,299.1 ± 966.4 for mini-GAGR) (Fig. 9, *A* and *B*). Conversely, the amount of Keap1 co-precipitated with Nrf2 was significantly decreased (∼73%) by mini-GAGR treatment compared with that by vehicle treatment (band density, 7775.6 ± 1387.4 for control *versus* 2073.2 ± 861.4 for mini-GAGR, *p* = 0.025) (Fig. 9, *A* and *C*). Thus, it is clear that mini-GAGR causes the dissociation of Nrf2 from Keap1 in the brains of 3xTg-AD mice intranasally treated with mini-GAGR.

Next, we examined whether intranasally administered mini-GAGR activates Nrf2 in the hippocampal neurons of 3xTg-AD mice by immunostaining p-Ser-40-Nrf2 in the hippocampal neurons of the mice treated with either vehicle or mini-GAGR as above. The sagittal sections (2–3 sections/animal, 6 animals/treatment group) of the hippocampal region of the mice were stained with antibodies to p-Nrf2 and NeuN (neuron marker) along with DAPI (Fig. 9, *D–G*). We confirmed the specificity of anti-p-Nrf2 antibody using mouse cortical neuron extracts (Fig. 9*H*). The CA1–CA3 and dentate gyrus (DG) regions of the hippocampi of mini-GAGR–treated 3xTg-AD mice (Fig. 9*F*) showed higher intensity of p-Nrf2 staining in the neurons compared with those of vehicle-treated 3xTg-AD mice (Fig. 9*D*). We looked closer at p-Nrf2 in hippocampal neurons in magnified images (Fig. 9, *E* and *G*). Compared with vehicle-treated 3xTg-AD mice (control; Fig. 9*E*), mini-GAGR-treated mice showed higher levels of p-Nrf2 in the nuclei in hippocampal CA1 neurons (Fig. 9*G*). We measured the intensities of the CA1–CA3 and DG regions and counted the number of p-Nrf2–positive neurons in the regions using Metamorph software for the calculation of average intensity ± S.E. and average number ± S.E. The intensity of p-Nrf2 was higher in the CA1–CA3 and DG regions of mini-GAGR–treated 3xTg-AD mice compared with vehicle-treated 3xTg-AD mice (-fold changes: CA1–CA3, 1.17 ± 0.08 for control *versus* 1.46 ± 0.12 for mini-GAGR (*p* = 0.0594); DG, 1.01 ± 0.05 for control *versus* 1.26 ± 0.07 for mini-GAGR (*p* < 0.01)) (Fig. 9*I*). Similarly, the number of p-Nrf2–positive neurons in the CA1–CA3 region was higher in mini-GAGR–treated 3xTg-AD mice compared with those with vehicle (-fold changes, 1 ± 0.18 for control *versus* 2.64 ± 0.16 for mini-GAGR, *p* < 0.001) (Fig. 9*J*). We confirmed that the increase in the number of p-Nrf2–positive neurons is not caused by the increase in cell mass in the region by hematoxylin and eosin (H&E) staining (Fig. S3*A*). In addition, we examined the levels of p-Nrf2 staining and p-Nrf2–positive neurons in WT mice (Fig. S3*B*). The p-Nrf2 intensity of hippocampal neurons in WT mice was higher than that of vehicle-treated 3xTg-AD mice (Fig. S3, *C* and *D*) (p-Nrf2 intensity in CA (1.62 ± 0.10-fold higher than vehicle (1.17 ± 0.08), *n* = 14, *p* = 0.006) as well as in DG (1.49 ± 0.10-fold higher than vehicle (1.01 ± 0.05), *n* = 14, *p* < 0.001) (Fig. S3*D*). p-Nrf2 intensity in WT mice was similar to that in mini-GAGR–treated 3xTg-AD mice (CA, *p* = 0.35; DG, *p* = 0.075). The numbers of p-Nrf2–positive neurons in WT mice were higher than those in vehicle-treated 3xTg-AD mice (2.64 ± 0.16-fold higher than vehicle (1 ± 0.18), *n* = 15, *p* < 0.001) (Fig. S3*E*). Conversely, the number of p-Nrf2–positive neurons in WT was comparable with that of mini-GAGR–treated 3xTg-AD mice (*p* = 0.99). Thus, it appears that mini-GAGR enters the brains of 3xTg-AD mice and activates Nrf2 in the hippocampus to a similar level as WT.

### Mini-GAGR reduces p-tau– and Aβ peptide–stained neurons and improves memory in 3xTg-AD mice

**Figure 10.**
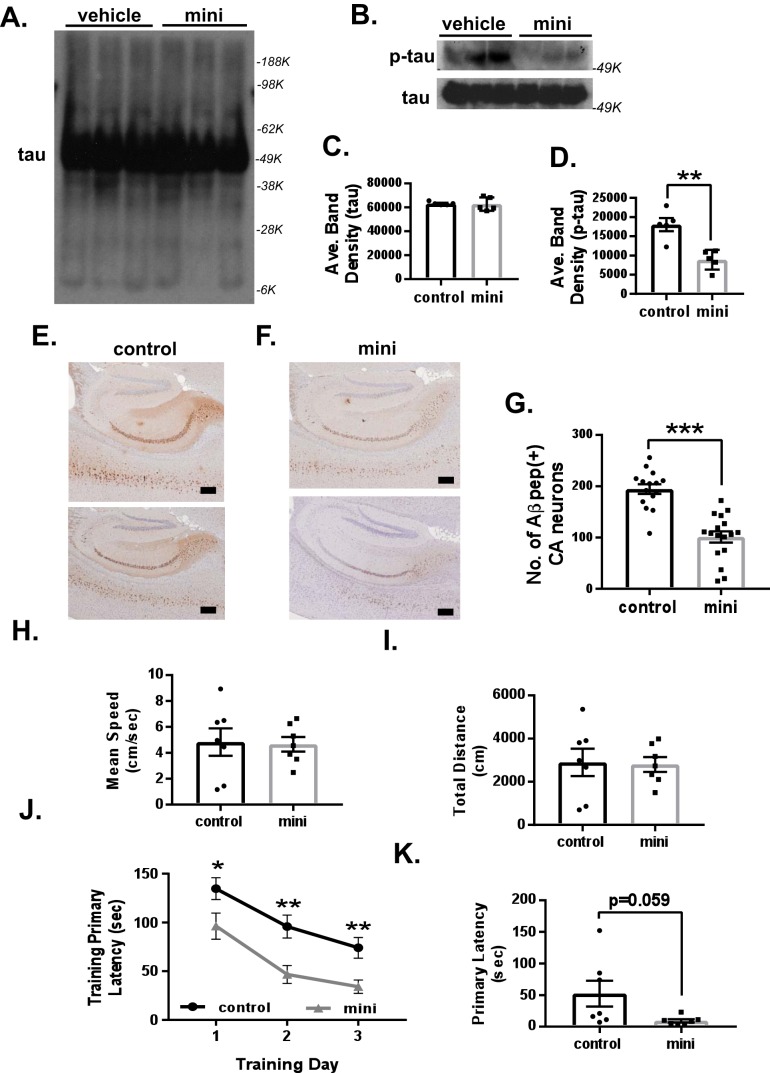
**Mini-GAGR reduces p-tau and Aβ peptide and improves learning and possibly memory retention in 12-month-old female 3xTg-AD mouse brains.** *A* and *B*, the cytosols of combined hippocampal and cortical tissues from 3xTg-AD mice treated with either vehicle or 100 nmol of mini-GAGR for 20 days were used for immunoblotting for p-tau and tau. The band densities of tau and p-tau were quantified by ImageJ to obtain average density ± S.E. (*error bars*) for bar graphs: tau (50 kDa): control *versus* mini-GAGR (*C*); p-tau (64 kDa): control *versus* mini-GAGR (*n* = 5 animals; **, *p* < 0.01) (*D*). *E* and *F*, the hippocampal sections of 3xTg-AD mice treated with either vehicle (*E*) or 100 nm mini-GAGR (*F*) were stained with anti-Aβ antibody (6E10) and HRP-tagged secondary antibody (*scale bar*, 250 μm). *G*, bar graphs show the average number of Aβ-positive hippocampal CA1-CA3 and DG regions of 3xTg-AD mice treated with either vehicle or 100 nm mini-GAGR (*n* = 6 animals (15 sections)/treatment; **, *p* < 0.01). *G–J*, 3xTg-AD mice treated with either vehicle or 100 nm mini-GAGR for 14 days were subjected to the open field paradigm to measure basal locomotor function. There was no difference in vehicle-treated *versus* mini-GAGR–treated mice regarding mean speed (cm/s) (*H*) and total distance (cm) (*I*). *J* and *K*, to determine the effect of mini-GAGR on learning and memory in 3xTg-AD mice (*n* = 7 mice/treatment group), the Barnes maze paradigm was performed. *J*, primary latency (s) of training days: vehicle-treated mice *versus* mini-GAGR–treated mice (*n* = 35 total training trials): training day 1 (*p* = 0.0309), training day 2 (*p* = 0.0016), and training day 3 (*p* = 0.00234). *K*, primary latency (s) of memory retention: vehicle-treated mice *versus* mini-GAGR–treated mice: memory retention (*p* = 0.0587) (*, *p* < 0.05; **, *p* < 0.01; ***, *p* < 0.001; unpaired *t* test, two-tailed). Data are expressed as mean ± S.E.

Given that oxidative stress is a major facilitator of AD pathogenesis, activating an Nrf2-dependent antioxidant defense system that reduces global oxidative stress (1) may attenuate AD pathogenesis, including the generation of p-tau and Aβ peptides. We briefly examined this possibility by looking at the protein levels of p-Ser-202-tau and Aβ peptide. First, we examined the protein levels of p-tau in the combined tissues of hippocampus and cortex by immunoblotting using the protein samples (*n* = 5/treatment group; vehicle *versus* mini-GAGR) used for Fig. 8. Whole cells were lysed in nucleus/cytosol extraction kit (Sigma) by 20 passages through a 27-gauge needle, spun at 500 × *g* to remove nuclei, and boiled in reducing SDS buffer prior to loading to protein gels. The result showed that the protein levels of p-tau were significantly reduced in mini-GAGR–treated 3xTg-AD mice compared with those in vehicle-treated 3xTg-AD mice (control), whereas those of total tau were not changed (band density: tau, 63,221.4 ± 821.3 for control *versus* 62,801.2 ± 3241.2 for mini-GAGR; p-tau, 18,048.1 ± 2213.3 for vehicle *versus* 8864.1 ± 1480.3 for mini-GAGR, *p* < 0.01) (Fig. 10, *B–D*). To see tau oligomer, we overexposed tau blots to film but could not detect an obvious difference in the amounts of tau oligomers between control and mini-GAGR (Fig. 10*A*). Thus, it appears that intranasally administered mini-GAGR significantly reduces p-tau in the hippocampus and its nearby cortex of 3xTg-AD mice.

Next, we examined the levels of Aβ peptides in the hippocampus and its nearby cortex of 3xTg-AD mice at 12 months of age by immunohistochemistry using anti-Aβ peptide primary antibody (6E10) and HRP-labeled secondary antibody (2–3 sections/animal, *n* = 6 animals/treatment group). The anti-Aβ peptide antibody (6E10) did not show any specific staining in the brains of WT mice (Fig. S4*A*) and was significantly decreased compared with 3xTg-AD mice (mean ± S.E.): 0 ± 0 (*n* = 15) for WT *versus* 194.5 ± 9.6 (*n* = 15) for 3xTg-AD, *p* < 0.001 (Fig. S4*B*). Compared with 3xTg-AD mice treated with vehicle (control) (Fig. 10*E*), those treated with mini-GAGR for 20 days appeared to accumulate fewer Aβ peptides in the hippocampus and its nearby cortex, whereas there was still a significant amount of Aβ peptides in the region in mini-GAGR–treated 3xTg-AD (Fig. 10*F*). We counted the number of Aβ peptide–positive neurons in the regions and found that the number of Aβ peptide-positive neurons was reduced in mini-GAGR–treated 3xTg-AD mice compared with vehicle-treated 3xTg-AD mice (194.47 ± 9.59 for vehicle *versus* 101.06 ± 10.94 for mini-GAGR, *p* < 0.001) (Fig. 10*G*). We also tried to stain amyloid precursor protein (APP) in these regions. Anti-APP antibody did not show any significant difference between WT, vehicle-treated, and mini-GAGR–treated 3xTg-AD mice (Fig. S4, *C* and *D*). The -fold change of the staining intensity of mean ± S.E. is as follows: 1 + 0.017 for WT, 1 ± 0.021 for 3xTg (control), and 1.02 ± 0.015 for mini-GAGR, *p* < 0.999, one-way ANOVA and Bonferroni's multiple-comparison test. Thus, it appears that intranasally administered mini-GAGR noticeably reduces Aβ peptides in the hippocampus and its nearby cortex of 3xTg-AD mice.

To briefly examine whether intranasally administered mini-GAGR improves learning and memory in 3xTg-AD mice at 12 months of age (7 animals/treatment group), we performed the Barnes maze, a known tool to test space-related memory. First, we performed the open field test to confirm no impaired locomotor activity in all treatment groups. In open field, there was no difference in mean speed (cm/s) (Fig. 10*H*) or total distance (cm) (Fig. 10*I*) between treatment groups (mean ± S.E.; mean speed, 4.83 ± 1.06 cm/s for control *versus* 4.66 ± 0.57 cm/s for mini-GAGR; total distance, 2901.08 ± 635.42 cm for control *versus* 2801.24 ± 340.95 cm for mini-GAGR). After testing in open field, 3xTg-AD mice were subjected to the Barnes maze to examine the effect of mini-GAGR on learning and memory. The mice had 3 days of training trials to test memory and a probe trial 24 h after, to test memory retention. In the Barnes maze, mice were placed at the center of the circular table containing 20 holes around the perimeter. Aversive stimuli (bright light and 100-db buzzer) were used to train the mice to enter the escape hole. We measured primary latency, which was the amount of time it took for the mouse to reach the target hole. In both training (Fig. 10*J*) and probe (Fig. 10*K*), mini-GAGR–treated mice appeared to have decreased primary latencies compared with vehicle-treated mice (control): primary latency: training day 1, 134.71 ± 11.20 s for control *versus* 96.23 ± 13.40 s for mini-GAGR, *p* < 0.05; training day 2, 95.74 ± 11.69 s for control *versus* 46.62 ± 9.20 s for mini-GAGR, *p* < 0.01; training day 3, 74 ± 10.63 s for control *versus* 34.03 ± 6.84 s for mini-GAGR, *p* < 0.01; memory retention, 52.29 ± 20.43 s for control *versus* 9.29 ± 2.52 s for mini-GAGR, *p* = 0.0587. Untreated age-matched WT mice (12 months old) were also tested in the Barnes maze. In training, WT mice had gradually shorter primary latencies compared with mini-GAGR–treated mice: primary latency, training day 1, 66.96 ± 10.99 s for WT *versus* 96.23 ± 13.40 s for mini-GAGR, *p* = 0.117; training day 2, 23.64 ± 5.44 s for WT *versus* 46.62 ± 9.20 s for mini-GAGR, *p* = 0.0548; training day 3, 13.4 ± 4.04 s for WT *versus* 34.03 ± 6.84 s for mini-GAGR, *p* < 0.05. In the probe, however, there was no significant difference between WT and mini-GAGR-treated 3xTg-AD mice: primary latency, memory retention, 6.2 ± 0.8 s for WT *versus* 9.29 ± 2.52 s for mini-GAGR, *p* = 0.343. In both training and probe, WT mice clearly demonstrated significantly shorter primary latencies during training compared with vehicle-treated 3xTg-AD mice (control): primary latency, training day 1, 66.96 ± 10.99 s for WT *versus* 134.71 ± 11.20 s for control, *p* < 0.001; training day 2, 23.64 ± 5.44 s for WT *versus* 95.74 ± 11.69 s for control, *p* < 0.001; training day 3, 13.4 ± 4.04 s for WT *versus* 74 ± 10.63 s for control, *p* < 0.001; memory retention, 6.2 ± 0.8 s for WT *versus* 52.29 ± 20.43 s for control, *p* < 0.05. These results suggest that intranasally administered mini-GAGR improves working memory (learning) and possibly memory retention in 12-month-old female 3xTg-AD mice to a level comparable with WT.

### Mini-GAGR increases PSD95 and GAP43 in the hippocampus and its nearby cortex in 3xTg-AD mice

**Figure 11.**
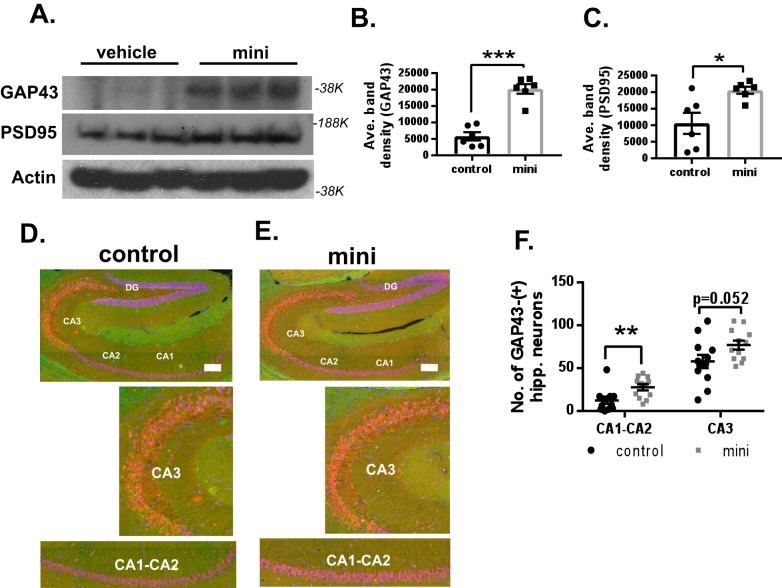
**Mini-GAGR increases PSD95 and GAP43 in the hippocampus and its nearby cortex in 12-month-old female 3xTg-AD mouse brains.** *A*, the cytosols of combined hippocampal and cortical tissues from 3xTg-AD mice treated with either 100 nmol of mini-GAGR for 20 days were used for immunoblotting for GAP43, PSD95, and actin. The band densities of GAP43 and PSD95 were quantified by ImageJ to obtain average density ± S.E. (*error bars*) for bar graphs: GAP43 (∼38 kDa) control *versus* mini-GAGR (*B*) and PSD95 (180 kDa) (*n* = 6 animals; *, *p* < 0.05; ***, *p* < 0.001) (*C*). *D* and *E*, hippocampal sections of 3xTg-AD mice treated with either vehicle (*D*) or 100 nm mini-GAGR (*E*) were stained with antibodies to GAP43 (*green*; Alexa Fluor 488) and NeuN (*red*; Alexa Fluor 594) and DAPI (*blue*). Displayed is the merge of this staining. *F*, scatter plots show the average number of GAP43-positive hippocampal CA1-CA2 and CA3 regions of 3xTg-AD mice treated with either vehicle or 100 nm mini-GAGR (*n* = 6 animals (12 sections)/treatment; **, *p* < 0.01) (*, *p* < 0.05; **, *p* < 0.01; ***, *p* < 0.001; unpaired *t* test, two-tailed). Data are expressed as mean ± S.E.

In our previous study (28), intranasally administered midi-GAGR increased the protein levels of post-synaptic density protein 95 kDa (PSD95) and growth-associated protein of 43 kDa (GAP43), two proteins indicating higher neuronal activity, in the hippocampi and cortices of 3xTg-AD mice. Compared with WT, PSD95 and GAP43 in vehicle-treated 3xTg-AD mice were decreased (Fig. S2). Average band density (mean ± S.E.) was as follows: PSD95, 26,589.0 ± 945.5 for WT *versus* 17,887.3 ± 1677.9 for vehicle-treated 3xTg-AD, *p* < 0.01; GAP43, 11,491.9 ± 1757.7 for WT *versus* 5778.85 ± 1255.63 for vehicle-treated 3xTg-AD, *p* < 0.01. Then we examined whether intranasally administered mini-GAGR also increases PSD95 and GAP43 in the brain region of 12-month-old 3xTg-AD mice by immunoblotting of proteins extracted from the region. Like midi-GAGR, mini-GAGR treatment increased both proteins statistically significantly in the combined tissues of hippocampi and cortices compared with vehicle treatment (Fig. 11, *A–C*). Average band density was as follows (means ± S.E.): GAP43, 5778.85 ± 1255.63 for control *versus* 20,183.43 ± 1458.12 for mini-GAGR, *p* < 0.001; PSD95, 10,565.6 ± 3186.6 for control *versus* 20,552.56 ± 1072.1 for mini-GAGR (*p* = 0.014). This result suggests that intranasally administered mini-GAGR activates a neurotrophic signaling pathway that enhances the expression of those proteins. Given that GAP43 is expressed in both neurons and glial cells (49), we examined whether the increase in GAP43 is specific to neurons by immunohistochemistry on hippocampal sections using antibodies to GAP43 (Alexa Fluor 488; *green*) and NeuN (Alexa Fluor 594; *red*). GAP43 staining in the hippocampus was weak but noticeable. We counted the number of GAP43-positive neurons in the hippocampus (*n* = 6 mice, 12 brain sections) and found that there was an increase in the number of GAP43-positive hippocampal neurons in mini-GAGR–treated 3xTg-AD mice compared with vehicle-treated (mean ± S.E.): 12.25 ± 3.76 for control CA1-CA2 *versus* 27.58 ± 3.53 for mini-GAGR CA1-CA2, *p* = 0.007; 57.83 ± 7.64 for control CA3 *versus* 76.92 ± 5.28 for mini-GAGR CA3, *p* = 0.052. As such, the intensity of GAP43 was increased in the hippocampal neurons of mini-GAGR–treated 3xTg-AD mice compared with that in vehicle-treated 3xTg-AD mice (Fig. 11, *D* and *E*). This result confirmed that intranasally administered mini-GAGR increased neuronal GAP43 in the hippocampus of 3xTg-AD mice (Fig. 11*F*). We also quantified the number of GAP43-positive hippocampal neurons in WT mice (Fig. S4, *E* and *F*). We found that mini-GAGR–treated 3xTg-AD mice had a slightly higher number of GAP43-positive CA3 neurons than WT mice (mean ± S.E.): 16.83 ± 4.56 for WT CA1-CA2 (*p* = 0.075 compared with mini-GAGR); 58.17 ± 6.14 for WT CA3 (*p* = 0.030 compared with mini-GAGR). Conversely, the number of GAP43-positive neurons in WT mice was similar to that of vehicle-treated 3xTg-AD mice (compared with vehicle-treated mice (12.25 ± 3.76), CA1-CA2, *p* = 0.45; CA3 vehicle (57.83 ± 7.64), *p* = 0.97) (Fig. S4*F*). Thus, it is clear that mini-GAGR has an ability to increase PSD95 and GAP43 (neurons) in the hippocampus of 3xTg-AD mice.

## Discussion

Nrf2 is considered a promising therapeutic target to control global oxidative stress in neurodegenerative brain diseases including AD because its activation initiates the expression of key endogenous antioxidant and detoxicating enzymes to counteract oxidative stress and regain redox homeostasis (1). In an AD model animal brain, Nrf2 is decreased in the nucleus (8), implying that Nrf2 loses its stance to activate the expression of antioxidant enzymes in the brain. In APP/PS1 AD model mice (7), overexpression of Nrf2 enhanced neuroprotection against Aβ peptides and improved spatial learning (7). In Tg19959 AD model mice, activating Nrf2 increased neuron survival against Aβ peptides by increasing antioxidant enzymes (50) and reduced oxidative stress, inflammation, and memory deficit (11). Thus, it appears that activating Nrf2 and its antioxidant defense system helps attenuate AD progression. Nonetheless, it is challenging to activate neuronal Nrf2 inside the brain because of the BBB. There are a few BBB-permeable Nrf2 activators, such as DMF and CDDO, found thus far, whereas their efficacy in slowing AD still needs further investigation.

In our previous study, we found a strong neuroprotective effect of midi-GAGR, a 4.7-kDa BBB-bypassing cleavage product (28, 29) of low-acyl gellan gum that is approved by the Food and Drug Administration as a human food additive and has few side effects in humans (27). Then how do the cleavage products of low-acyl gellan gum exert neuroprotective effects? Given that the antioxidant enzyme system is the major endogenous defense against oxidative stress, we hypothesized that the cleavage products of low-acyl gellan gum, mini-GAGR and midi-GAGR, activate the neuronal antioxidant defense system for protection against oxidative stress. To test our hypothesis, first, we examined the effect of mini-GAGR on the protein levels of major antioxidant enzymes in mouse embryonic cortical neurons (E17, DIV11–14). Under resting conditions, 48-h treatment with 1 μm mini-GAGR increased protein levels of HO-1, SOD1, GR, and GPx4, four major antioxidant enzymes, without changing those of GAPDH and β-actin (Fig. 1), suggesting that mini-GAGR is able to increase antioxidant enzyme proteins in the neurons. Next, given that Nrf2 is the major activator of the antioxidant enzyme system (1), we speculated that mini-GAGR might activate Nrf2. 3-h treatment with 1 μm mini-GAGR significantly increased not only the nuclear localization of Nrf2 but also its transcriptional activity (Fig. 2), suggesting that mini-GAGR is an Nrf2 activator. We also found that the efficacy of mini-GAGR in increasing Nrf2 nuclear localization and transcriptional activation was comparable with midi-GAGR and other Nrf2 activators (DMF (4) and CDDO-TFEA (45, 46)) that are already in clinical trials (DMF for multiple sclerosis, CDDO-TEFA (RTA-408) for Huntington's disease and Friederich's ataxia) (Fig. 3). In our unpublished results,^4^ because 0.1 μm MIND4, another Nrf2 activator (51), caused the death of 50% of mouse cortical neurons after 2-day incubation, it was not further tested. All four Nrf2 activators except MIND4 increased protein levels of SOD1 and GPx4 to higher levels than control after 48-h treatment. Of note, there was a variation in their acute and chronic effects on Nrf2 and its downstream enzyme proteins, implying that different Nrf2 activators appear to use different mechanisms to activate Nrf2. Our results suggest that mini-GAGR can activate Nrf2 and its downstream to an extent comparable with other Nrf2 activators examined.

Because the antioxidant defense system should work in the presence of oxidative stress in AD brains, we examined whether mini-GAGR could increase antioxidant enzymes in mouse cortical neurons in the presence of pathological concentrations of 4HNE and H_2_O_2_. The presence of either 4HNE or H_2_O_2_ did not change the protein levels of antioxidant enzymes compared with control (Fig. 4). Pretreatment with mini-GAGR showed only a trend of increase in the protein levels of most antioxidant enzymes in the presence of 4HNE but not H_2_O_2_. Intriguingly, the protein levels of HO-1 were significantly increased by the co-presence of mini-GAGR and free reactive radicals, suggesting that HO-1 expression may be controlled by an alternative mechanism to the other tested antioxidant enzymes. For example, in endothelial cells, CREB boosts Nrf2-driven expression of HO-1 (52). Given that midi-GAGR activates CREB (28), mini-GAGR is expected to activate CREB as well, which likely further activates HO-1 expression with Nrf2. Unexpectedly, NQO1, a known Nrf2 downstream protein, was not increased in response to mini-GAGR. These intriguing results of NQO1 and HO-1 suggest that mini-GAGR may use a nonclassical Nrf2-ARE system to activate the expression of antioxidant enzymes.

In addition, we examined the effects of mini-GAGR on the enzymatic activity of SOD and the intracellular levels of ROS and lipid peroxide (MDA) in neurons exposed to either 4HNE or H_2_O_2_. Mini-GAGR alone significantly increased SOD activity and did not affect intracellular levels of ROS and MDA in neurons. The increase in SOD enzymatic activity appears to be more significant than that in its protein level in response to mini-GAGR, suggesting that mini-GAGR may use an unknown intracellular system to further increase SOD activity. In addition, mini-GAGR lowered the intracellular levels of ROS and MDA in neurons that were exposed to 4HNE, suggesting that mini-GAGR can lower ROS and lipid peroxide in neurons exposed to 4HNE. Conversely, the reducing effect of mini-GAGR on ROS and MDA in H_2_O_2_-exposed neurons, however, did not reach statistical significance. This suggests that the free radical–reducing effect of mini-GAGR is specific to 4HNE.

In our study, 4HNE and H_2_O_2_ slightly increased the intracellular level of ROS, whereas only 4HNE slightly increased that of MDA. The reason why 4HNE and H_2_O_2_ did not cause significant increases in ROS and MDA appear to be due to ∼25% loss of neurons (28) that are excluded from free radical measurements. A similar reason may explain why 4HNE and H_2_O_2_ did not drastically change the protein levels of antioxidant enzymes as we loaded the same amounts of proteins from remaining cells (∼75%) to those of control and mini-GAGR–treated neurons for immunoblotting. Even with the same amounts of proteins used, we could still detect an improvement in the enzymatic activity of SOD1 and an increase in HO-1 in mini-GAGR–treated neurons.

At early-stage AD, mitochondrial disruption starts with the uncoupling of the mitochondrial electron transport chain and the depolarization of MMP, which results in ROS production and ATP depletion (25, 26, 53). Therefore, without protecting mitochondria, it is not possible to reduce ROS production and neurodegeneration. Both 4HNE and H_2_O_2_ that were used for ROS and MDA experiments drastically disturbed mitochondrial integrity (MitoTracker) and MMP (JC-1) (Fig. 5), suggesting that mitochondria respond more sensitively to the pathological concentrations of 4HNE and H_2_O_2_ than ROS and MDA production. Because mini-GAGR treatment enhances Nrf2 and its antioxidant enzyme system, it is possible that mini-GAGR reduces mitochondrial disruption and MMP loss caused by free reactive radicals. As expected, mini-GAGR treatment restored the intensity of MitoTracker and JC-1 back to control levels in neurons exposed to either 4HNE or H_2_O_2_, suggesting that mini-GAGR protects mitochondria from oxidative stress. Thus, it is clear that mini-GAGR has an ability to preserve mitochondrial integrity and MMP in mouse cortical neurons under oxidative stress.

Because the extent of mini-GAGR-mediated restoration of mitochondria and MMP is extensive, we speculate that reducing oxidative stress alone may not be the only mechanism used by mini-GAGR to maintain functional mitochondria under oxidative stress. It is possible that mini-GAGR also activates additional Nrf2 downstream that up-regulates the expression of genes involved in mitochondrial biogenesis via Nrf1 and mitochondrial transcription factors (TFAM) (54), thus rejuvenating dying mitochondria in neurons under oxidative stress. This possible effect of mini-GAGR on mitochondria will be studied in our next study.

Our shRNA study confirmed that the mini-GAGR–induced increase in antioxidant enzymes depended on Nrf2. With anti-Nrf2 shRNA, there was a ∼30% knockdown of Nrf2 compared with control shRNA–transfected neurons for both vehicle and mini-GAGR treatment conditions (Fig. S1). The 30% knockdown of Nrf2 appears to be sufficient to reduce the increase in HO-1 and GPx4 and the trend of increase in CAT and GR in response to mini-GAGR treatment (Fig. S1). These data suggest that the mini-GAGR–induced increase in protein expression of HO-1 and GPx4 (and possibly CAT and GR) depends on Nrf2.

To elucidate the mechanism by which mini-GAGR activates Nrf2, we revisited the inhibitory interaction of Keap1 with Nrf2 (14), the PKC-dependent activation of Nrf2 (55), and the involvement of FGFR1 (28) in mini-GAGR–dependent Nrf2 activation. Our data showed that mini-GAGR treatment caused the dissociation of Nrf2 from Keap1 in mouse cortical neurons as evident in colocalization and co-immunoprecipitation results (Fig. 6). We also found that mini-GAGR increased the Ser-40 phosphorylation of Nrf2 that is known to be mediated by PKC (55) and increased the enzymatic activity of PKC, which was blocked by staurosporine, a known PKC inhibitor (Fig. 7). Staurosporine also blocked mini-GAGR–induced Ser-40 phosphorylation and nuclear localization of Nrf2, indicating that PKC is involved in mini-GAGR–induced activation and nuclear localization of Nrf2 in mouse cortical neurons. Given that mini-GAGR and midi-GAGR share the same basic unit, it is possible that FGFR1 is involved in mini-GAGR–mediated Nrf2 activation. As expected, FGFR1 inhibitor blocked mini-GAGR–induced Ser-40 phosphorylation and nuclear localization of Nrf2 in mouse cortical neurons, suggesting that FGFR1 plays a role in mini-GAGR–mediated Nrf2 activation. Our finding of the involvement of FGFR1 in the activation of the antioxidant system is consistent with previous findings (56–58). Thus, our results indicate that mini-GAGR causes dissociation of Nrf2 from Keap1 and Ser-40 phosphorylation of Nrf2 in a PKC- and FGFR1-dependent manner.

Our solid *in vitro* data led us to a rationale to pursue the examination of the effects of mini-GAGR in an AD animal model. First, we confirmed that mini-GAGR could enter the brain via intranasal administration that is used to bypass the BBB for some BBB-impermeable compounds (59). Like midi-GAGR (28, 29), 0.2 μm mini-GAGR accumulated inside the brains of 10-month-old WT mice after its intranasal administration (Fig. 8, *A* and *B*). We cannot specify how mini-GAGR bypasses the BBB at this point, although it is possible that intranasally administered mini-GAGR may enter the brain via the olfactory and trigeminal nerves, the cerebrospinal fluid, and/or the lymphatic system (60).

As it did in WT mice, mini-GAGR was expected to enter the brain of 11-month-old 3xTg-AD mice more easily because the BBB of 3xTg-AD mice was reported to be less tight (61), similarly to BBB breakdown observed in post-mortem human AD brains (62). Indeed, intranasally administered mini-GAGR increased the expression of antioxidant enzymes such as HO-1, SOD1, and GPx4 (Fig. 8) to similar levels in WT mice (Fig. S2) as it did *in vitro*. After examination of the WT mice compared with the 3xTg-AD mice, it was evident that the significant decreases in antioxidant enzymes, catalase (Fig. S2*E*) and HO-1 (Fig. S2*B*), and the trending decreases (SOD, *p* = 0.065; GPx, *p* = 0.071) appeared to be transgenic-dependent. Mini-GAGR treatment was able to restore the transgenic-dependent decreases and trends of these antioxidant enzymes of 3xTg-AD mice back to WT levels (Fig. 8). Specifically, it did so in the cortex and hippocampus that are implicated and compromised in AD (63). In line with the increase in the expression of antioxidant enzymes, we observed Nrf2 activation in the hippocampi of 3xTg-AD mice. The dissociation of Nrf2 from Keap1 and the levels of p-Nrf2 (Ser-40) were increased in the brains and hippocampal neurons, respectively, of mini-GAGR–administered 3xTg-AD mice. In contrast to mini-GAGR–treated 3xTg-AD mice, the levels of p-Nrf2 were weakly detected in the brains of WT mice and vehicle-treated 3xTg-AD mice, which is in accordance with a previous report (64). In other words, Nrf2 is epigenetically repressed in adult neurons (64). Thus, it is clear that intranasally administered mini-GAGR activates Nrf2 in the hippocampal neurons.

In addition to Nrf2 and its downstream antioxidant enzymes, intranasally administered mini-GAGR also increased the protein levels of GAP43 and PSD95 in the hippocampus and cortex of 3xTg-AD mice. Because glial cells also express GAP43, we confirmed that the increase of GAP43 is specific to hippocampal neurons by immunohistochemistry. As expected from the fact that GAP43 is typically down-regulated with brain maturation except in the CA3 hippocampus as well as regions of the brainstem (65), GAP43 staining was weak in neurons in CA1–CA3 and DG regions while detectable in CA3 hippocampal neurons. Even with the weak staining, we could still find more neurons that have stronger GAP43 signals in mini-GAGR–treated 3xTg-AD mice than those in water-treated 3xTg-AD mice.

The dual effects of mini-GAGR, namely Nrf2-activating and neurotrophic effects, should provide mini-GAGR with an advantage over other Nrf2 activators that have little neurotrophic effect in attenuating AD progression. Given that Nrf2 and its antioxidant defense system lose their stance against globally increased oxidative stress in AD brains, restoring redox balance by activating Nrf2 and its antioxidant defense system should be beneficial to reduce global oxidative stress in AD brain. In addition, activating Nrf2 should have other beneficial effects (*e.g.* attenuating inflammation (66) and activating autophagy (67)) in mitigating AD pathogenesis. Along with the Nrf2-activating effect, its neurotrophic effect is expected to slow neurodegeneration by mimicking neurotrophic factors that enhance synaptogenesis and neuronal activity (33, 68–78) and reduce neurodegenerative factors, such as p-tau (79–81). Indeed, 20-day intranasal treatment with mini-GAGR reduced the protein levels of p-tau (p-Ser-202) as well as the number of Aβ peptide-stained neurons in the hippocampus of 12-month-old 3xTg-AD mice (Fig. 10) that develop AD hallmarks such as high levels of p-tau and Aβ peptides around 12 months of age (39). Thus, mini-GAGR is capable of exerting multimodal effects that attenuate AD pathogenesis; those include Nrf2-activating, neurotrophic, and p-tau– and Aβ–reducing effects.

Around 12 months of age, 3xTg-AD mice, especially female mice, were reported to develop memory deficit (39), whereas some studies showed minor memory defects in 12-month-old 3xTg-AD mice (43, 82, 83). This suggests that variability exists in individual 3xTg-AD mice regarding memory-related behaviors and sensitivity to given experimental conditions. Nonetheless, in our Barnes maze study, we could observe obvious defects in working memory (learning) and memory retention in 12-month-old 3xTg-AD mice treated with water compared with their WT mice. As expected from its multimodal AD-attenuating effects, mini-GAGR treatment improved working memory significantly during 3-day training and memory retention noticeably in 3xTg-AD mice compared with water treatment (Fig. 10). The shortened primary latency in mini-GAGR–treated 3xTg-AD mice was comparable with that of WT mice, specifically in the memory retention test (*p* = 0.343). This suggests that mini-GAGR is able to restore memory retention close to WT level. Because our treatment period is relatively short, it was not expected to drastically reduce AD hallmarks and memory deficit in 3xTg-AD mice that undergo AD pathogenesis for at least 9 months. Nonetheless, with the short time period of treatment, mini-GAGR could generate noticeable changes in those. Therefore, it is likely that a longer treatment such as 4–6 months from an earlier age would have more drastic attenuating effects on AD pathogenesis in 3xTg-AD mice.

The AD-attenuating effect of mini-GAGR in 3xTg-AD mice is comparable with other Nrf2 activators. Gracilin A was reported to reduce Aβ_42_ and p-tau and produce a positive trend of an improvement in primary latency at 24 h after the final training in 3xTg-AD mice during the Morris water maze (13). DMF was also reported to reduce p-tau via Akt-GSK3β activation in the hippocampus of mice transfected with TAUP301L-overexpressing adenovirus (84). Carnosic acid, a pro-electrophilic Nrf2 activator, decreased Aβ_42_ and p-tau in hAPP-J20 mice (85). Hydrogen sulfide also reduced Aβ peptides and improved memory in APP/PS1 mice (86). There are, however, Nrf2 activators that cannot affect Aβ_42_ and p-tau. For example, methysticin could not alter Aβ burden in the hippocampus of APP/Psen1 mice while activating Nrf2 and reducing microgliosis, astrogliosis, inflammation, and memory deficit (87). Collectively, AD-attenuating effects of mini-GAGR are comparable with the precedent Nrf2 activators in reducing Aβ_42_ and p-tau, whereas it has an additional neurotrophic effect.

To this end, we found a BBB-bypassing Nrf2 activator, mini-GAGR, in the human food additive low-acyl gellan gum, which has few side effects in humans. Like low-acyl gellan gum, mini-GAGR is expected to have few side effects in humans. Indeed, animals treated with mini-GAGR did not show any abnormalities in sleeping, drinking, eating, grouping, locomotor activity, weight, and other daily activities during our studies, indicating that mini-GAGR has no harmful effect on animals. Our next step will be identifying the exact mechanism by which mini-GAGR activates Nrf2 signaling and the neurotrophic pathway, which will help us to speculate about its possible off-target effects. At the current stage, it is hard to claim a therapeutic advantage of mini-GAGR and midi-GAGR over other Nrf2 activators (*e.g.* DMF and CDDO) that are already in clinical trials. Nonetheless, we believe that the dual ability of mini-GAGR to activate simultaneously the Nrf2-ARE system and neurotrophic signaling pathway certainly provides a better chance to control multifaceted AD.

## Experimental procedures

### Materials

Low-acyl gellan gums (>99% pure) were obtained from CPKelco Co. (Atlanta, GA) and dissolved in milliQ H_2_O for enzymatic digestion. 0.6 g of low-acyl gellan gum (CPKelco) was incubated in 200 ml of milliQ H_2_O containing 1% salicin, 0.05 m sodium acetate (pH 5.0 adjusted with acetic acid) with 10 mg of the recombinant protein, α(1→3)-glycosidase (Agn1 (MyBioSource)), for 72 h to produce mini-GAGR. The enzyme reaction was stopped by incubation in a hot water bath for 5 min and dried in a vacuum dryer. Dried gel pellet was then washed extensively in de-ionized water by stirring for 48 h (freshwater replaced every 12 h) to wash off salts and enzyme from the pellet and prepared in the matrices, α-cyano-4-hydroxycinnamic acid and 2,5-dihydroxybenzoic acid, on an MS plate. The samples were assessed for both direct and reflected laser paths in a Bruker 500 mass spectrometer. Midi-GAGR was generated by 48-h digestion (29, 88). H_2_O_2_ was purchased from Sigma, 4HNE from Cayman Chemical (Ann Arbor, MI), ANTS from Molecular Probes/Life Technologies, Inc. (Eugene, OR), DMF from Sigma-Aldrich, CDDO-TFEA from Cayman Chemical (Ann Arbor, MI), MitoTracker and JC-1 from Life Technologies (Carlsbad, CA), staurosporine from Sigma, and PD173074 from Sigma. Protease and phosphatase inhibitors were purchased from Thermo Scientific (Rockford, IL).

### Primary cell culture

Female pregnant mice (BALB/c, E17, Charles River Laboratories International, Inc.) were anesthetized and dissected to obtain 6–8 embryos/animal. Cortical neurons were isolated from embryonic brains and differentiated for 11–14 days *in vitro* (DIV11–14) on poly-l-lysine–coated bottom or coverslips in 6-well and 96-well plates containing Dulbecco's modified Eagle's medium (Life Technologies) plus 10% fetal bovine serum plus Primocin (Invitrogen). A hemocytometer was used to load an equal number of neurons per well (6-well plate, 1.87 × 10^6^ cells/well; 96-well plate, 0.234 × 10^4^ cells/well). On the following day, Dulbecco's modified Eagle's medium was replaced with B27/neurobasal medium plus Primocin. For immunocytochemistry, neurons (DIV11–14) on coverslips were treated with either vehicle (H_2_O) or 1 μm mini-GAGR for 0, 1, 3, 6, or 24 h for the nuclear localization of Nrf2 using primary antibodies to Nrf2 and βIII tubulin and DAPI and for 0 and 3 h for the colocalization assay using primary antibodies to Keap1 and Nrf2. To examine the effects of Nrf2 activators on nuclear Nrf2 localization and Nrf2 phosphorylation, neurons were treated with vehicle, 1 μm mini-GAGR, 1 μm midi-GAGR, 6 μg/ml DMF, or 100 nm CDDO-TFEA for 3 h prior to either the staining using primary antibodies to Nrf2 and βIII tubulin and DAPI or the immunoblotting using primary antibodies to Nrf2 and p-Nrf2. To examine the involvement of PKC and FGFR1 in mini-GAGR–mediated Ser-40 phosphorylation of Nrf2, neurons were pretreated with vehicle, 3 nm staurosporine, or 50 nm PD173074 for 2 h and treated with either vehicle or 1 μm mini-GAGR prior to immunoblotting (Nrf2 and p-Nrf2) and immunocytochemistry (Nrf2, βIII tubulin, and DAPI). For the staining of MitoTracker (a mitoprobe for mitochondrial membrane integrity), neurons were treated with either vehicle (H_2_O) or 1 μm mini-GAGR for 16 h and with vehicle, 10 μm 4HNE, or 50 μm H_2_O_2_ for 30 h prior to 30-min incubation with 0.3 μm MitoTracker at 37 °C, fixation in 3.7% formaldehyde, and mounting on slides. For the staining of JC-1 (a mitoprobe for monitoring mitochondrial health), neurons were seeded in a polylysine-coated live cell chamber (Delta TPG dish, 0.17-mm clear, Fisher Scientific) and treated in the same way as for MitoTracker prior to 30-min incubation with 2 μm JC-1 at 37 °C and live cell imaging. To inhibit PKC and FGFR1 prior to mini-GAGR treatment, neurons were pretreated with vehicle, 3 nm staurosporine, or 50 nm PD173074 for 2 h, treated with 1 μm mini-GAGR for 3 h, and then processed for either immunostaining using antibodies to Nrf2 and βIII tubulin and DAPI or immunoblotting for p-Ser-40-Nrf2, Nrf2, and PKC. To examine the effects of mini-GAGR, midi-GAGR, DMF, and CDDO-TFEA on the protein levels of SOD1 and GPx4, neurons were treated with vehicle, 1 μm mini-GAGR, 1 μm midi-GAGR, 6 μg/ml DMF, or 100 nm CDDO-TFEA for 48 h prior to protein extraction for immunoblotting.

### Immunoblotting and immunocytochemistry

For immunoblotting, neurons were harvested from 6-well plates using trypsin + EDTA (Sigma) and lysed in 1% Igepal CA-630 (Sigma)–containing PMEE buffer (89) and protease inhibitor mixtures (and phosphatase inhibitor if phosphoproteins were being detected) for protein extraction. 10 μg of extracted proteins were loaded into each well of a 15-well 4–11% NuPAGE protein gel (Life Technologies), separated on the basis of molecular weight, and transferred to polyvinylidene difluoride membrane (Invitrogen) using a wet transfer (Life Technologies). Protein bands on blots were recognized by primary antibodies (1:1000) and HRP-conjugated secondary antibody (1:10,000) and detected on Amersham Biosciences Hyperfilm ECL films (GE Healthcare) with SuperSignal West Pico Chemiluminescent Substrate (Thermo Scientific). The antibodies used for immunoblotting (and estimated molecular masses) were as follows: Abcam Biotech (Cambridge, MA) (anti-Nrf2 (80 kDa) (ab31163, rabbit), anti-p-Ser-40-Nrf2 (100 kDa) (ab76026, rabbit), anti-PKC (81 kDa) (ab23511, mouse), anti-SOD1 (16 kDa) (ab13498, rabbit), anti-βIII tubulin (ab78078, mouse), anti-APP (ab15272, rabbit), and NeuN (ab104224, mouse)); Santa Cruz Biotechnology (anti-Keap1 (70 kDa) (sc-365626, mouse), anti-β-actin (45 kDa) (sc-69879, mouse), anti-CAT (64 kDa) (sc-50508, rabbit), anti-GR (60 kDa) (sc-133245, rabbit), anti-GPx4 (21 kDa) (sc-166570, rabbit), anti-HO-1 (32 kDa) (sc-136960, mouse), and anti-GAPDH (37 kDa) (sc-32233, mouse)); Cell Signaling Technology (Danvers, MA) (anti-NQO1 (29 kDa) (3187S, mouse)); Thermo Fisher Scientific (anti-pS202-tau (64 kDa) (MN1020B, mouse)); Biolegend (San Diego, CA) (anti-Aβ (6E10, SIG-39320, mouse), anti-GAP43 (38 kDa) (sc-17790, mouse), and anti-PSD95 (180 kDa) (sc-32290, mouse). The densities of protein bands on films were quantified using ImageJ software. For immunocytochemistry, neurons after different treatments were fixed in 3.7% paraformaldehyde in PBS for 30 min, permeabilized in 0.1% Triton X-100, and blocked in 2% BSA and 0.1% Tween in TBS (B-TBS-T). Then neurons on coverslips were stained with primary antibodies (1:100, 30 min), secondary Alexa Fluor 488 or Alexa Fluor 594 antibody (1:1000, 15 min (DAPI)) in a wet chamber. Coverslips were mounted on glass slides using Fluoromount G (Fisher). Images of neurons were taken using a TCS SP5 multiphoton laser-scanning confocal microscope (Leica Microsystems, Bannockburn, IL). After imaging, the intensities of Nrf2 and MitoTracker in the nuclei were quantified using Metamorph Software (Molecular Probes/Life Technologies). The extents of the colocalization between Keap1 and Nrf2 were quantified using ImageJ.

### Enzyme and chemical assays

The kits were prepared in accordance with the manufacturers' instructions. Primary cortical mouse neurons for enzyme assays were harvested using cold buffer (PBS + EDTA) and a cell scraper and processed as follows.

#### 

##### Transcriptional activity of Nrf2 (Abcam)

To measure the transcriptional activity of Nrf2, we used the Nrf2 Transcription Factor Assay kit (Colorimetric) (catalog no. ab207223). Neurons in a 6-well plate were treated with vehicle, 1 μm mini-GAGR, 1 μm midi-GAGR, 6 μg/ml DMF, or 100 nm CDDO-TFEA for 3 h and harvested in PBS buffer containing phosphatase and protease inhibitor mixtures (Thermo Scientific). Neurons were lysed on ice in hypotonic buffer containing (20 mm Hepes, pH 7.5, 0.1 mm EDTA with phosphatase inhibitors) and 10% Igepal CA-630 to obtain nuclear pellet. Nuclei were further extracted in Complete Lysis Buffer (containing 1 mm DTT and 1% protease inhibitor mixture). Nuclear extracts were transferred to a 96-well plate of the assay kit and incubated to allow the binding of Nrf2 to its consensus sequence on DNA in the 96-well plate, followed by primary anti-Nrf2 antibody and secondary HRP antibody. Nonspecific binding was removed by extensive wash, added with developing solution prior to absorbance measurement at 450 nm.

##### SOD (Cayman Chemical)

Total SOD activity (units/ml) was determined using the SOD Assay Kit (catalog no. 706002). Samples were prepared according to the manufacturer's instructions. After neurons were treated with either vehicle or mini-GAGR (1 μm) and free radical treatment (vehicle, 10 μm 4HNE, or 50 μm H_2_O_2_) as described under “Primary cell culture,” the neurons were used for SOD measurement. A standard curve was generated using various concentrations of bovine erythrocyte SOD (Cu/Zn). A radical detector containing tetrazolium salt solution, assay buffer, and sample was mixed and added to the wells of a 96-well plate. After the rapid addition of xanthine oxidase to the wells and incubation at room temperature, the absorbance of each well was measured at 450 nm and used to calculate SOD activity (units/ml).

##### ROS (Cayman Chemical)

ROS generation in live neurons was assessed by the quantification of DHE, a fluorescent probe (RFU), using the ROS Detection Cell-Based Assay Kit (catalog no. 601290). Neurons were seeded in a black-wall 96-well plate. After neurons were treated with either vehicle or mini-GAGR (1 μm) and free radical treatment (vehicle, 10 μm 4HNE, or 50 μm H_2_O_2_) as described under “Primary Cell Culture,” the neurons were washed with assay buffer, and ROS-staining buffer containing dihydroethidium was added to the wells. The plate was incubated at 37 °C for 1.5 h in the dark, and after a final wash with assay buffer, fluorescence was measured at excitation of 500 nm and emission of 585 nm.

##### Lipid peroxidation (MDA) (Abcam)

MDA, a product of lipid peroxidation, was assessed using the Lipid Peroxidation (MDA) Assay Kit (catalog no. ab118970). A standard curve was generated with various concentrations of MDA (μm). After neurons were treated with either vehicle or mini-GAGR (1 μm) and free radical treatment (vehicle, 10 μm 4HNE, or 50 μm H_2_O_2_) as described under “Primary cell culture,” the neurons were harvested, washed, and lysed in MDA lysis buffer with BHT and homogenized on ice. Standards and samples were vigorously boiled for 1 h with TBA solution containing glacial acetic acid to generate an MDA-TBA adduct. Following an ice bath and centrifugation, supernatant solution containing MDA-TBA adduct was transferred to a 96-well plate, and fluorescence was measured at excitation/emission of 532/553 nm.

##### PKC kinase activity (Abcam)

To measure PKC kinase activity, we used the PKC Kinase Activity Assay Kit (catalog no. 139437). Neurons in a 6-well plate were pretreated with either vehicle or 3 nm staurosporine for 2 h and treated with either vehicle or mini-GAGR (1 μm) for 3 h. Then the neurons were harvested in lysis buffer (20 mm HEPES, 5 mm EGTA, 2 mm EDTA, 1% Nonidet P-40, 1 mm DTT, protease and phosphatase inhibitor mixtures). Supernatant was collected and diluted in Kinase Assay Dilution Buffer. Reactions were initiated with the addition of ATP and followed by PKC phosphospecific substrate antibody incubation and anti-rabbit IgG-HRP conjugate incubation with thorough washes between the steps. Detection was performed using TMB substrate, and absorbance was measured at 450 nm.

### Nrf2 knockdown using shRNA

To determine whether Nrf2 is required for mini-GAGR–mediated increases in antioxidant enzyme proteins, we knocked down Nrf2 using Nrf2 shRNA (m: anti-mouse Nrf2) lentiviral particles. Mouse cortical neurons (E17, DIV8) were transfected with 50,000 inclusion-forming units/1.87 × 10^6^ cells of either control shRNA or Nrf2 shRNA for 1 day, incubated in fresh media for 5 days, and then treated with either vehicle or 1 μm mini-GAGR for 2 days prior to protein extraction for immunoblotting.

### Animal treatment

3-Month-old 3xTg-AD female mice (*B6; 129-Psentm1Mpm Tg* [*APPSwe, tauP301L*] *1Lfa/Mmja)x*) were purchased from Jackson Laboratory (Bar Harbor, ME) and aged until the age of 11 months prior to the examination of the effect of mini-GAGR. 3xTg-AD mice demonstrate only some aspects of human AD pathology but are still the AD animal model closest to human AD. After 20-day treatment, seven 3xTg-AD mice per treatment (either mini-GAGR or water) were used for protein assays (immunoblotting and immunoprecipitation), and another six animals (3xTg-AD mice) per treatment (either mini-GAGR or water) were used for immunohistochemistry using antibodies to p-Nrf2/NeuN/DAPI, Aβ peptide (6E10), H&E, APP, and GAP43/NeuN/DAPI. The WT mouse colony is a mix of C57BL/6J and B6:129-Psen1tm1Mpm and has the same genetic background as 3xTg-AD mice. These WT mice were established by breeding together mixed genetic backgrounds C7BL/6;129X1/SvJ;129S1/Sv. After arrival at the Jackson Laboratory, interbreeding was done for 17 generations; prior to that remains unknown (strain 004807, stock no. 34830). Six WT mice were used for protein assays and six WT mice for immunohistochemistry. Eight WT female mice at 10 months of age were from the Jackson Laboratory and used for the examination of the BBB permeability of mini-GAGR tagged with ANTS (4 mice for water and 4 mice for ANTS–mini-GAGR). Animals were housed at room temperature under a 12-h light/dark cycle. Food and water were provided *ad libitum*. All of the procedures of animal use outlined in this study were approved by the animal care and use committee of the University of Toledo College of Medicine and Life Science in accordance with National Institutes of Health guidelines. All experiments were performed during the light phase (7 a.m. to 7 p.m.). To examine the ability of mini-GAGR to enter the brain, 100 nmol/40 μl mini-GAGR conjugated to ANTS (29) was intranasally administered into 10-month-old WT mice (*n* = 4). 6 h later, the mice were anesthetized by intraperitoneal injection of ketamine and xylazine and sacrificed via cervical dislocation. The amounts of fluorescent ANTS–mini-GAGR in the cytosol of whole brains were quantified by measuring the fluorescence of ANTS using a microplate reader (29). A standard curve was generated with 0.1–0.5 mm ANTS-conjugated mini-GAGR. The values of ANTS fluorescence in the brain cytosols were converted to the concentrations of mini-GAGR using the standard curve. To examine the effect of BBB-bypassing mini-GAGR on the protein levels of antioxidant enzymes, p-tau, and Aβ peptides in the hippocampi and cortices of 3xTg-AD mice and their behavior, we intranasally administered 100 nmol/40 μl mini-GAGR into the nostrils of the mice (average weight = 25.5 g) once per day for 14 days. Thereafter, mice were tested for animal behavior for 5 days prior to euthanization after 20-day treatment (mice were continuously treated during behavior tests until euthanization). Then brains were extracted from euthanized mice to obtain the hippocampi and their neighboring cortices, which were either used immediately or snap-frozen on dry ice for later use. Brain tissues were homogenized in 1× PMEE buffer containing 1% Igepal CA-630 and protease inhibitor mixture with a 27-gauge syringe needle. The homogenization was incubated on ice for 30 min at 4 °C and then centrifugation at 13,000 × *g* for 30 min for cytosol extraction. 15 μg of extracted proteins were loaded into each well of 15-well 4–11% NuPAGE gel. For immunohistochemistry, six WT mice, six 3xTg-AD mice treated with mini-GAGR, and six 3xTg-AD mice treated with vehicle were anesthetized with a combination of ketamine/xylazine and perfused with a wash of PBS (40 ml was used to remove contaminating blood) followed with transcardial perfusion of 4% paraformaldehyde prior to the extraction of their brains for another 24-h fixation in 4% formalin. Then serial 15-μm sagittal sections were generated using a freezing microtome, treated in 3% H_2_O_2_/methanol and in 80% formic acid, incubated in TBS + 0.1% Triton X-100 + 5% BSA and in primary antibodies and secondary antibodies (either fluorescent (Alexa Fluor 488 or Alexa Fluor 594) or HRP). Stained sections were mounted on slides and imaged using a VS 120 Virtual Slide Microscope (Olympus Life Science, Waltham, MA).

### Animal behavior testing

#### 

##### Open field

12-Month-old 3xTg-AD female mice treated with either vehicle or mini-GAGR for 14 days were used in the behavioral testing. Animals were treated continuously during behavior tests until the 20th day from the start of treatment. Seven mice per treatment with either water or mini-GAGR represented four mice used for protein assays plus three mice used for immunohistochemistry after 20-day treatment. Mice were brought into the testing room in their home cages and allowed to acclimate for 10 min. The room was kept quiet with the light on throughout experiments. Panlab SMART video tracking software, Smart version 3.0, was utilized to record and analyze the testing trials. Open field tests were done in a 71.92 × 71.92-cm chamber that was separated into a grid of 36 squares. This chamber was thoroughly cleaned with diluted 70% ethanol before and after each trial. After setting up the software and cleaning the chamber, mice were tested individually. Each mouse was removed from its home cage and gently placed into the chamber at the center of the grid. Mice were allowed to explore for 10 min and were then returned to their home cage. Each trial was recorded for later analysis.

##### Barnes maze

Barnes maze testing was done on a circular 92-cm diameter white table with 20 5-cm-diameter holes spaced evenly along the perimeter. The circular platform was mounted on the top of a rotating stool, 95 cm above the ground. The escape cage was made of a black plastic box directly below the surface of the maze. The location of the maze stayed the same throughout the testing and was surrounded by false walls to prevent mice from seeing the observers. Simple colored-paper shapes (squares, triangles, circles) were mounted onto the walls surrounding the maze, serving as visual cues. A weighted down black cylindrical cup was used to contain mice at the center of the maze to allow the mice to face in random directions at the start of each trial. After each session, the maze, escape cage, and the cup were all cleaned with diluted 70% ethanol. The mice underwent 3 days of training trials in which there were 5 trials/day. In the training phase, mice were placed at the center of the maze underneath the weighted cylindrical black cup. After 10 s, the chamber was lifted, and the buzzer (100 db) and lights were turned on. Mice were allowed to explore the maze for 3 min. The training trials ended when the mouse entered into its target escape hole, at which point the buzzer and lights were turned off and the mouse sat for 1 min. If the mouse did not make it into the target hole after 3 min had elapsed, they were guided to the escape cage via a clear cylindrical container, after which they were allowed to sit inside for 1 min with the buzzer and lights turned off. The mice were tested consecutively with cleaning of the maze, escape cage, and cups after each trial, allowing for an intertrial interval of about 20 min for each individual mouse. Each mouse received five trials during each of the three training days for a total of 15 training sessions. The probe phase took place 24 h after the last training session to measure memory retention. Learning and memory were assessed with the measurement of primary latency, or the time taken to the escape cage.

### Co-immunoprecipitation

For co-immunoprecipitation studies, neurons in seven 6-well plates were treated with either vehicle or 1 μm mini-GAGR for 3 h, harvested using trypsin + EDTA, and lysed in 1 ml of PMEE buffer containing 0.5% Igepal CA-630 and the inhibitors of proteases and phosphatases using a 27-gauge needle (10 triturations) and incubated on ice for 30 min for complete lysis. The cortices of 3xTg-AD mice treated intranasally for 19 days with either vehicle or 100 nmol of mini-GAGR were lysed in 2 ml of PMEE buffer containing 0.5% Igepal CA-630 and the inhibitors of proteases and phosphatases in the same way as above. Both neuron and brain lysates were spun at 13,000 × *g* for 30 min to obtain cytosol. 1 ml of the cytosol fraction (0.5 mg/ml protein) was split into two sets of 500 μl. Each 500 μl of the cytosol was mixed with either 5 μg of control rabbit IgGs or rabbit IgGs against Nrf2 and incubated for 18 h at 4 °C. Then 60 μl of protein A–agarose beads were added to the tubes and incubated for 8 h. After incubation, the beads were washed seven times with PMEE buffer and boiled in 100 μl of SDS loading buffer. Proteins were separated by NuPAGE gel (Invitrogen), transferred to polyvinylidene difluoride membrane, and detected by immunoblotting.

### Statistical analysis

Neuron culture experiments were replicated multiple times with different batches of cell cultures. Microscopic analysis was performed blindly. Colocalization was analyzed using the JACoP program of ImageJ to measure Pearson's coefficient *R*. The analysis for the IHC slices was done with Olympia OlyVIA 2.9 and Metomorph software. Animal behavior open field and Barnes maze videos were recorded and analyzed using Panlab SMART video tracking software, Smart version 3.0. Statistical significance between two groups was calculated using unpaired Student's t test, with a *p* < 0.05 confidence interval considered statistically significant. Multiple comparisons were performed using one-way ANOVA using a multiple-comparison test (GraphPad Prism version 7.04). F-tests (ANOVA) were used to assess equality of means and are reported as F(DFn, DFd). Data were tested for normality via the Shapiro–Wilk normality test (GraphPad Prism version 7.04).

## Author contributions

J. P. conceived the idea for the project, conducted the experiments, analyzed the results, and wrote the paper. K. M. conducted the majority of the experiments, analyzed the results, and contributed to experimental manuscript writing. K. L., S. W., M. F., J. P., and N. S. contributed the performance of the experiments and the analyses of the results. K. H. and D. K. contributed to the idea and the experimental designs.

## Supplementary Material

Supporting Information

## Footnotes

4 K. Murphy, K. Llewellyn, S. Wakser, J. Pontasch, N. Samanich, M. Flemer, K. Hensley, D.-S. Kim, and J. Park, unpublished results.

## Footnotes

3 The abbreviations used are:

AD: Alzheimer's disease
BBB: blood–brain barrier
DMF: dimethyl fumarate
ARE: antioxidant response element
SOD: superoxide dismutase
CAT: catalase
GR: GSH reductase
GPx: GSH peroxidase
γ-GCS: γ-glutamyl cysteine synthetase
NQO1: NAD(P)H:quinoneoxidoreductase 1
MMP: mitochondrial membrane potential
ROS: reactive oxygen species
PKC: protein kinase C
FGFR: fibroblast growth factor receptor
Aβ: amyloid β
4HNE: 4-hydroxynonenal
E: embyronic day
DIV: day(s) in vitro
GAPDH: glyceraldehyde-3-phosphate dehydrogenase
DAPI: 4′,6-diamidino-2-phenylindole
HRP: horseradish peroxidase
CDDO: 2-cyano-3,12-dioxooleana-1,9(11)-dien-28-oic acid
TFEA: trifluoroethylamide
DHE: dihydroethidium
MDA: malondialdehyde
RFU: relative fluorescence units
IP: immunoprecipitation
Stau: staurosporine
PD: PD173074
DG: dentate gyrus
p-: phosphorylated
H&E: hematoxylin and eosin
CA: cornu ammonis
APP: amyloid precursor protein
ANOVA: analysis of variance
CREB: cAMP-response element–binding protein.
