# Supplementary material for: Mini-GAGR, an intranasally applied polysaccharide, activates the neuronal Nrf2-mediated antioxidant defense system
Source: J Biol Chem. 2018 Oct 3;293(47):18242–69. doi: 10.1074/jbc.RA117.001245 (PMC6254342; doi:10.1074/jbc.RA117.001245)
Supplement: Supporting Information [file supp_293_47_18242__index.html]

Mini-GAGR, an intranasally applied polysaccharide, activates the neuronal Nrf2-mediated antioxidant defense system — BBB-bypassing Nrf2 activator — Mini-GAGR, an intranasally applied polysaccharide, activates the neuronal Nrf2-mediated antioxidant defense system — BBB-bypassing Nrf2 activator — Supporting Information 

# Mini-GAGR, an intranasally applied polysaccharide, activates the neuronal Nrf2-mediated antioxidant defense system

## Supporting Information

- Supplemental Data - Figs S1-S4
