# Supplementary material for: Mini-GAGR, an intranasally applied polysaccharide, activates the neuronal Nrf2-mediated antioxidant defense system
Source: J Biol Chem. 2018 Oct 3;293(47):18242–69. doi: 10.1074/jbc.RA117.001245 (PMC6254342; doi:10.1074/jbc.RA117.001245)
Supplement: Supporting Information [file supp_RA117.001245_134346_3_supp_210486_pfr33z.pdf]

Supplementary Figure Legends

Figure S1. Knockdown of Nrf2 blocks mini-GAGR-induced increases in HO-1 and GPx4. *(A)* Mouse cortical neurons (E17, DIV8) were transfected with 50,000 IFU/1.87 x 10^6 cells of either control shRNA or anti-Nrf2 shRNA for 5 days and treated with either vehicle (control: con) or 1 μM mini-GAGR for 2 days prior to immunoblotting using antibodies to Nrf2, β-actin, HO-1, SOD1, CAT, GR, and GPx4. The band densities of antioxidant enzyme proteins were quantified by Image J to obtain Ave. density ± SEM for bar graphs. No statistically significant differences between control shRNA conditions for *(B)* Nrf2 (80kD), *(D)* SOD1 (16kD), *(E)* CAT (64kD), and (F) GR (60kD). Significant differences between control shRNA conditions for *(C)* HO-1 (32kD) and *(G)* GPx4 (21kD): (*: $p<0.05$, students t-test). Data are expressed as mean± SEM.

Figure S2. The comparison of the protein levels of antioxidant enzymes in the brains of 12-month-old wild type vs. 3xTg-AD mice (vehicle-treated). *(A)* The brain cytosols of 3xTg-AD 12-month-old wild type (WT) and 3xTg-AD (3xTg) mice (vehicle-treated) were used for immunoblotting using antibodies to antioxidant enzymes. The band densities of antioxidant enzymes in the cytosols were quantified by Image J to obtain Ave. density±SEM for bar graphs. *(B)* HO-1 (32kD): control vs. mini-GAGR (*: $p<0.05$), *(C)* SOD1 (16kD): control vs. mini-GAGR, *(D)* GPx4 (21kD): control vs. mini-GAGR, *(E)* CAT (64kD): control vs. mini-GAGR (***: $p<0.001$), *(F)* NQO1 (31kD): control vs. mini-GAGR, *(G)* GAP43: control vs. mini-GAGR, *(H)* PSD95: control vs. mini-GAGR ($p<0.01$), *(I)* β-actin (45kD): control vs. mini-GAGR. (*: $p<0.05$; **: $p<0.01$; ***: $p<0.001$, unpaired t test, two-tailed). Data are expressed as mean±SEM.

Figure S3. H&E staining and p-Nrf2 staining in wild type and 3xTg-AD mice treated with either vehicle or mini-GAGR. *(A)* The H&E-stained hippocampal sections of 3xTg-AD mice (3xTg) treated with either vehicle (control) or 100 nM mini-GAGR (mini) for 20 days and wild type (WT) mice (scale bar = 250 μm). The hippocampal sections of *(B)* wild type mice and *(C)* 3xTg mice (vehicle-treated) were stained with antibodies to p-Nrf2 (green, Alexa488) and NeuN (red, Alexa598) along with DAPI (blue) (scale bar = 250 μm). *(D)* Scatter plots show the fold changes of intensities of p-Nrf2 staining in hippocampal CA1-CA3 and DG regions of WT or 3xTg-AD mice (vehicle-treated) (n=6 animals [14 sections] per phenotype, **: $p<0.01$; ***: $p<0.0001$). *(E)* Bar graphs show the fold changes of p-Nrf2-positive hippocampal CA1-CA3 regions of WT mice or 3xTg-AD mice (vehicle-treated) (n=6 animals [15 sections] per phenotype, *: $p<0.05$; **: $p<0.01$; ***: $p<0.001$; unpaired t test, two tailed). Data are expressed as mean±SEM.

Figure S4. Aβ, APP, and GAP43 staining in wild type and 3xTg-AD mice treated with either vehicle or mini-GAGR. *(A)* The hippocampal sections of wild type (WT) mice and 3xTg-AD (3xTg) mice (vehicle-treated) were stained with anti-Aβ antibody (6E10) and HRP-tagged secondary antibody (scale bar = 250 μm). *(B)* Bar graph shows the Ave. number (mean±SEM) of Aβ-positive hippocampal CA regions of WT or 3xTg mice (vehicle-treated) (n=6 animals [15 sections] per treatment, ***: $p<0.001$). *(C)* The hippocampal sections of 3xTg-AD mice treated with either vehicle or 100 nM mini-GAGR for 20 days and wild type mice were stained with anti-APP antibody and HRP-tagged secondary antibody (scale bar = 250 μm). *(D)* Bar graphs show the fold changes of the intensities of APP staining in hippocampal CA1-CA3 of WT, vehicle-treated 3xTg-AD, or mini-GAGR-treated 3xTg-AD mice (n=6 animals [10-14 sections] per phenotype/treatment, not significant, one-way ANOVA and Bonferroni's multiple comparisons test, [$F(2, 35) = 0.22932$, $p=0.796$]. *(E)* The hippocampal sections (CA1-CA2 and CA3) of wild type mice and 3xTg mice were stained with antibodies to GAP43 (green, Alexa488) and NeuN (red, Alexa598) and DAPI (blue). The merge of this staining is displayed. *(F)* Scatter plots show the Ave. number (mean±SEM) of GAP43-positive hippocampal CA1-CA2 and CA3 regions of WT and 3xTg-AD

mice (n=6 animals [12 sections] per treatment). (*: $p<0.05$; **: $p<0.01$; ***: $p<0.001$, unpaired t test, two tailed). Data are expressed as mean±SEM.

Figure S1

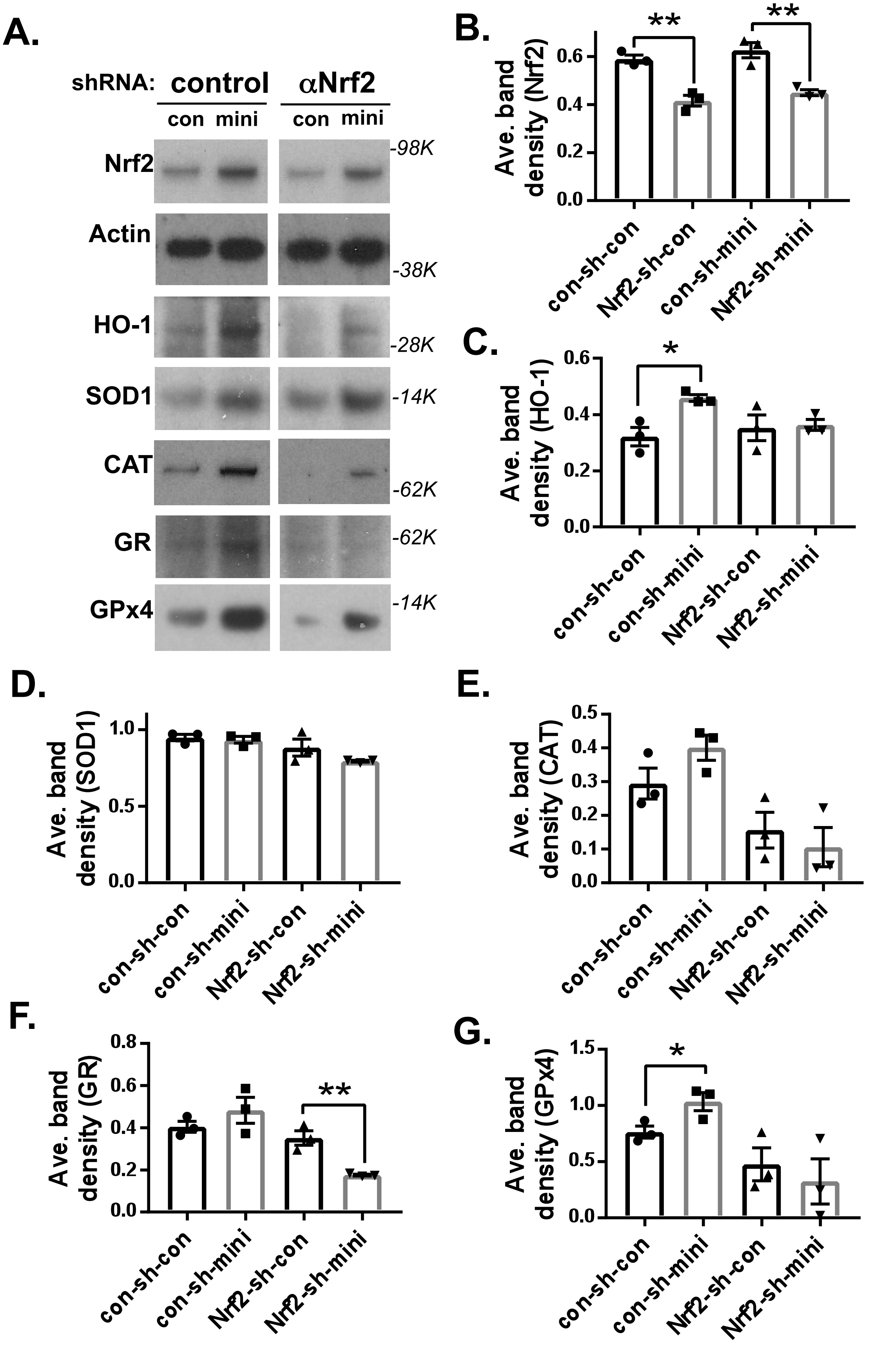
A.
shRNA: control αNrf2
con mini con mini
Nrf2
Actin
HO-1
SOD1
CAT
GR
GPx4
-98K
-38K
-28K
-14K
-62K
-62K
-14K
B.
Ave. band density (Nrf2)
con-sh-con
Nrf2-sh-con
con-sh-mini
Nrf2-sh-mini
C.
Ave. band density (HO-1)
D.
Ave. band density (SOD1)
E.
Ave. band density (CAT)
F.
Ave. band density (GR)
G.
Ave. band density (GPx4)

Figure S2

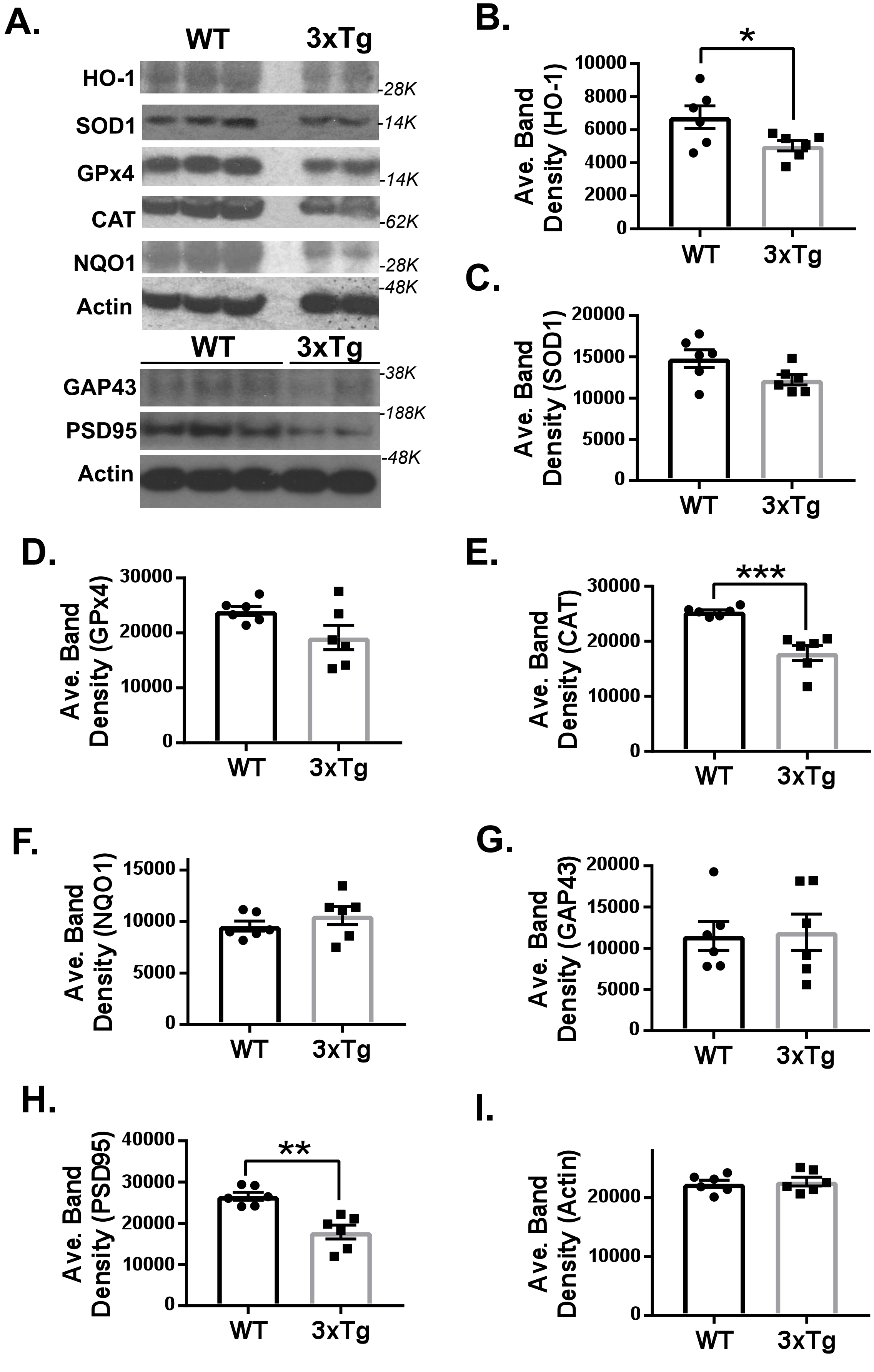

A.
WT
3xTg
HO-1
SOD1
GPx4
CAT
NQO1
Actin
GAP43
PSD95
Actin
-28K
-14K
-14K
-62K
-28K
-48K
-38K
-188K
-48K
B.
C.
D.
E.
F.
G.
H.
I.
Ave. Band Density (HO-1)
Ave. Band Density (SOD1)
Ave. Band Density (GPx4)
Ave. Band Density (CAT)
Ave. Band Density (NQO1)
Ave. Band Density (GAP43)
Ave. Band Density (PSD95)
Ave. Band Density (Actin)
*
***
**

Figure S3

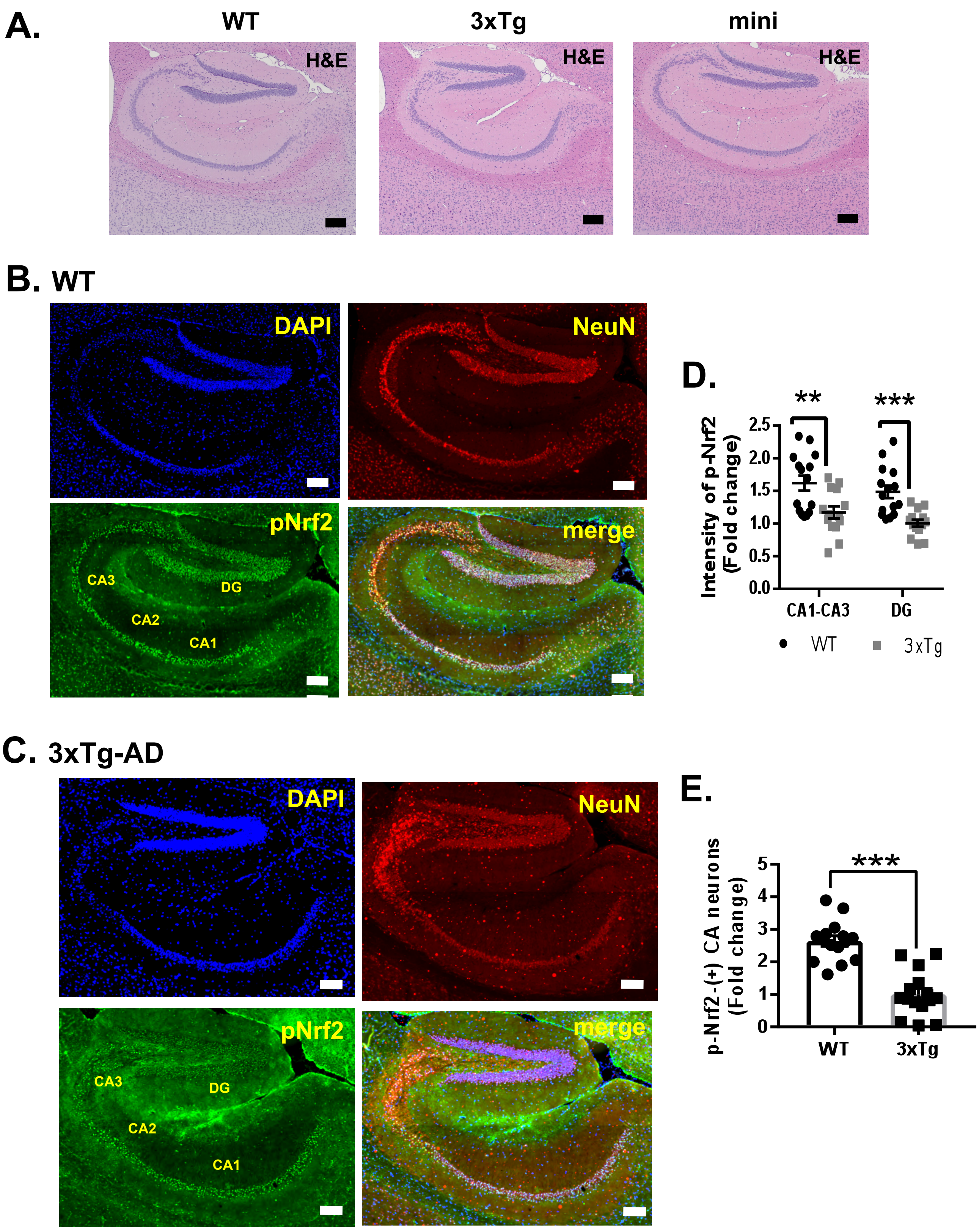

A.
WT
3xTg
mini
H&E
H&E
H&E
B. WT
DAPI
NeuN
pNrf2
merge
CA3
DG
CA2
CA1
C. 3xTg-AD
DAPI
NeuN
pNrf2
merge
CA3
DG
CA2
CA1
D.
**
***
Intensity of p-Nrf2 (Fold change)
2.5
2.0
1.5
1.0
0.5
0.0
CA1-CA3
DG
WT
3xTg
E.
***
p-Nrf2-(+) CA neurons (Fold change)
5
4
3
2
1
0
WT
3xTg

Figure S4

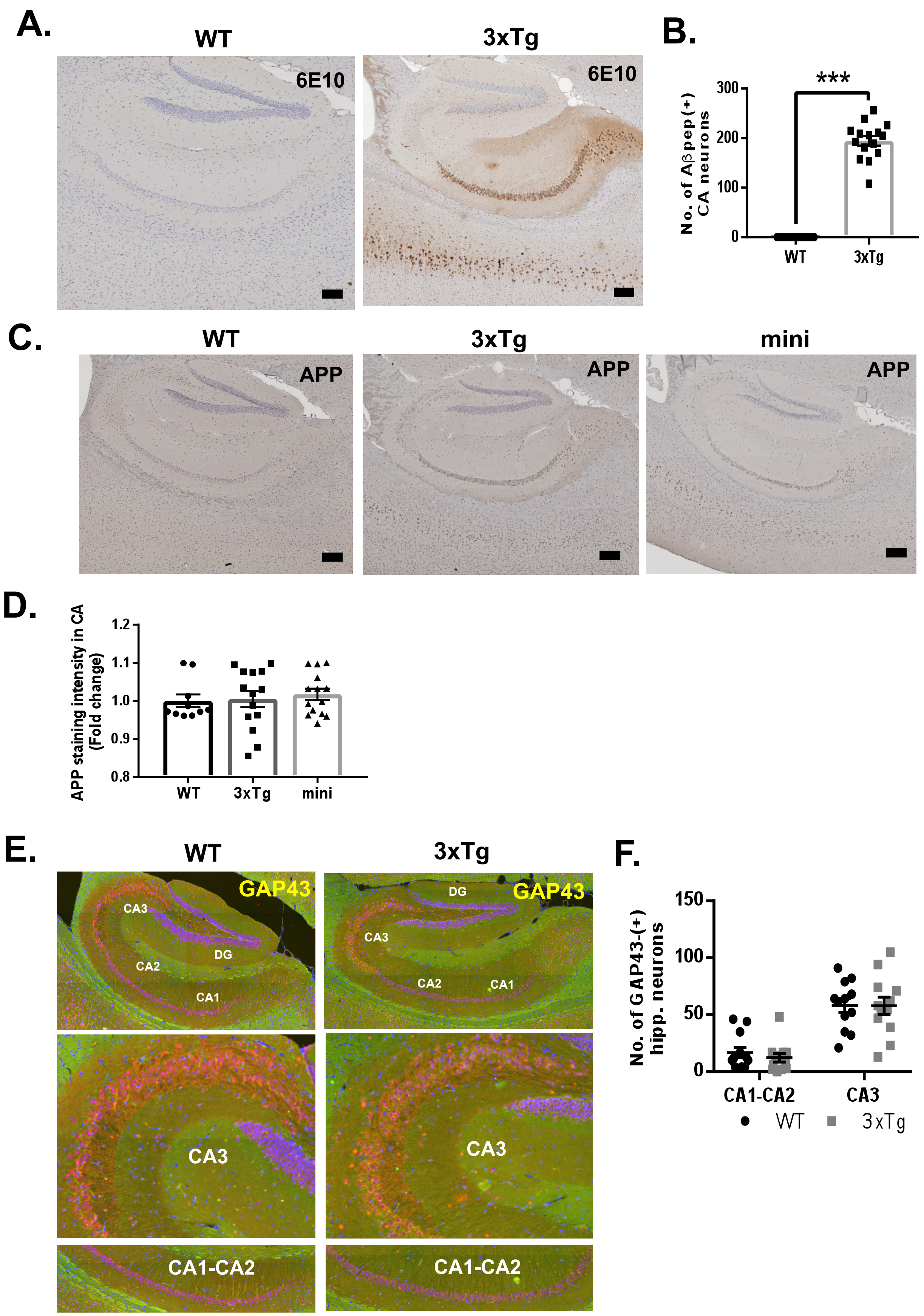
A.
WT
3xTg
6E10
B.
***
No. of Aβpep(+) CA neurons
300
200
100
0
WT
3xTg
C.
WT
3xTg
mini
APP
D.
APP staining intensity in CA (Fold change)
1.2
1.1
1.0
0.9
0.8
WT
3xTg
mini
E.
WT
3xTg
GAP43
CA3
CA2
CA1
DG
CA1-CA2
F.
No. of GAP43-(+) hipp. neurons
150
100
50
0
CA1-CA2
CA3
WT
3xTg
